# Plasmid-mediated colistin resistance from fresh meat and slaughtered animals in the Czech Republic: nation-wide surveillance 2020–2021

**DOI:** 10.1128/spectrum.00609-23

**Published:** 2023-09-12

**Authors:** Petra Sismova, Iva Sukkar, Nikita Kolidentsev, Jana Palkovicova, Ivana Chytilova, Jan Bardon, Monika Dolejska, Kristina Nesporova

**Affiliations:** 1 Department of Biology and Wildlife Diseases, Faculty of Veterinary Hygiene and Ecology, University of Veterinary Sciences Brno, Brno, Czech Republic; 2 Central European Institute of Technology, University of Veterinary Sciences Brno, Brno, Czech Republic; 3 State Veterinary Institute, Prague, Czech Republic; 4 Department of Microbiology, Faculty of Medicine and Dentistry Palacky University Olomouc, Olomouc, Czech Republic; 5 State Veterinary Institute Olomouc, Olomouc, Czech Republic; 6 Department of Clinical Microbiology and Immunology, Institute of Laboratory Medicine, University Hospital Brno, Brno, Czech Republic; 7 Biomedical Centre, Faculty of Medicine in Pilsen, Charles University, Pilsen, Czech Republic; Forschungszentrum Jülich GmbH, Juelich, Germany

**Keywords:** screening, colistin, resistance, *mcr*, livestock

## Abstract

The aim of this study was to determine the occurrence of plasmid-mediated colistin resistance in domestic and imported meat and slaughter animals in the Czech Republic during 2020–2021 by using selective cultivation and direct PCR testing. A total of 111 colistin-resistant *Escherichia coli* isolates with *mcr-1* gene were obtained from 65 (9.9%, *n* = 659) samples and subjected to whole-genome sequencing. Isolates with *mcr* were frequently found in fresh meat from domestic production (14.2%) as well as from import (28.8%). The *mcr-1*-positive *E. coli* isolates predominantly originated from meat samples (16.6%), mainly poultry (27.1%), and only minor part of the isolates came from the cecum (1.7%). In contrast to selective cultivation, 205 (31.1%) samples of whole-community DNA were positive for at least one *mcr* variant, and other genes besides *mcr-1* were detected. Analysis of whole-genome data of sequenced *E. coli* isolates revealed diverse sequence types (STs) including pathogenic lineages and dominance of ST1011 (15.6%) and ST162 (12.8%). Most isolates showed multidrug-resistant profile, and 9% of isolates produced clinically important beta-lactamases. The *mcr-1* gene was predominantly located on one of three conjugative plasmids of IncX4 (83.5%), IncI2 (7.3%), and IncHI2 (7.3%) groups. Seventy-two percent isolates of several STs carried ColV plasmids. The study revealed high prevalence of *mcr* genes in fresh meat of slaughter animals. Our results confirmed previous assumptions that the livestock, especially poultry production, is an important source of colistin-resistant *E. coli* with the potential of transfer to humans via the food chain.

**IMPORTANCE:**

We present the first data on nation-wide surveillance of plasmid-mediated colistin resistance in the Czech Republic. High occurrence of plasmid-mediated colistin resistance was found in meat samples, especially in poultry from both domestic production and import, while the presence of *mcr* genes was lower in the gut of slaughter animals. In contrast to culture-based approach, testing of whole-community DNA showed higher prevalence of *mcr* and presence of various *mcr* variants. Our results support the importance of combining cultivation methods with direct culture-independent techniques and highlight the need for harmonized surveillance of plasmid-mediated colistin resistance. Our study confirmed the importance of livestock as a major reservoir of plasmid-mediated colistin resistance and pointed out the risks of poultry meat for the transmission of *mcr* genes toward humans. We identified several *mcr*-associated prevalent STs, especially ST1011, which should be monitored further as they represent zoonotic bacteria circulating between different environments.

## INTRODUCTION

Resistance to last-line antimicrobials shows a steady increase in the last decade in both human and animal sectors. One of these critically important antimicrobials is colistin (polymyxin E), acting on a wide range of Gram-negative bacteria (1). Although the use of colistin in humans has been very limited in the past, nowadays it has been recovered for the treatment of serious systemic infections caused by multidrug-resistant (MDR) bacteria (2). Whereas colistin usage was seriously limited in human medicine, it was broadly administered in veterinary medicine, especially in poultry and pigs, for the prevention and therapy of commonly occurring gastrointestinal infections caused by bacteria from Enterobacterales (3, 4).

Despite its medicinal importance, colistin is still added to animal feed as a growth promoter for livestock in some countries, contributing significantly to the global spread of colistin resistance (4). European Union (EU) countries have restricted the prophylactic use of colistin-containing veterinary products, including a ban on all antimicrobials as growth promoters in animal feed, as early as 2006 (4). In the Czech Republic, colistin consumption in veterinary medicine decreased by 25.0% between 2010 and 2018 to 0.7 milligrams/population correction unit (mg/PCU). The EU has defined a target to reduce colistin usage below 1 mg/PCU; however, some European countries such as Spain, Portugal, and Hungary with consumption above 10 mg/PCU exceed this value (5).

Colistin resistance can be associated with mutations in specific chromosomally encoded genes or mediated by *mcr* genes carried mainly by plasmids. Plasmid-mediated colistin resistance was first discovered in 2015 (1) and so far, 10 *mcr* gene variants (*mcr-1* to *mcr-10*) with several subvariants have been identified in Enterobacterales, predominantly in *Escherichia coli* (6 – 8). Globally, the most prevalent variant of the *mcr* gene-encoding colistin resistance is *mcr-1*. The gene has been found in several sources including humans, animals, agricultural products, and the environment, indicating a high capacity for spread across different sectors (6, 9).

Plasmid-mediated colistin resistance is commonly transferred by conjugative plasmids of various incompatible groups (Inc) including IncX4, IncI2, and IncHI2 types (10). Some *mcr*-carrying isolates show resistance to cephalosporins, carbapenems, and other antibiotics, whose resistance genes may be localized within the same plasmid as *mcr* (11) posing a significant risk because of possible transfer of resistance to last-line antibiotics in a single conjugative event.

The discovery of the transferable mechanism created the urge to monitor the level to which colistin critically functions as the last resort antibiotics against MDR bacteria is compromised. The spread of *mcr* genes and the development of colistin resistance are monitored by national antibiotic resistance surveillance programs including the European Antimicrobial Resistance Surveillance Network sponsored by the European Centre for Disease Prevention and Control (12). In the Czech Republic, a study aimed at detecting *mcr* genes from human clinical isolates has been ongoing in collaboration with the National Reference Laboratory for Antibiotics since 2018 (13).

Although colistin consumption is limited in many countries, an increased incidence of plasmid-mediated colistin resistance in bacteria isolated from food animals, especially poultry and pigs, has been reported (9). The results indicate that the veterinary sector is a likely source of *mcr* genes, thereby contributing to the global spread of colistin resistance (14). The objectives of this study were to determine the occurrence of plasmid-mediated colistin resistance in fresh meat samples and slaughtered animals from the Czech Republic as a part of nation-wide surveillance program and to characterize the isolates and plasmids carrying *mcr* genes using whole-genome sequencing (WGS).

## MATERIALS AND METHODS

### Sampling and selective cultivation

This study involved a total of 659 samples of domestic and imported meat and cecal samples from slaughtered animals obtained between May 2020 and May 2021. The collection consisted of retailed fresh meat samples from domestic (46.0%) and foreign production (9.0%) including poultry (*n* = 181), pork (*n* = 91), and beef (*n* = 90), and cecum samples (45.0%) from slaughtered poultry (*n* = 183) and pigs (*n* = 114). Details regarding sampling can be found in the Supplementary Material and Methods. All types of samples (meat and cecum) were homogenized, and a small amount was used and incubated in 3 mL of buffered peptone water (BPW) overnight. The following day, 9 mL of Luria-Bertani broth (LBB) with colistin (1 mg/L) was added to the sample of BPW, and the mixture was cultivated overnight. Enriched samples were subcultivated on eosin methylene blue (EMB) agar and MacConkey agar (MCA) containing colistin (3.5 mg/L) to obtain colistin-resistant isolates. One presumptive isolate of *E. coli* collected from each agar plate was species identified by MALDI-TOF mass spectrometry (microflex LT/SH; Bruker Daltonics, Bremen, Germany) and subjected to isolation of DNA by thermal lysis. Moreover, 1 mL of the LBB-enriched samples was used for isolation of the whole-community DNA using NucleoSpin Tissue Isolation Kit (Macherey-Nagel, Germany).

### Detection of *mcr* genes

The presence of *mcr* genes (*mcr-1* to *mcr-10*) was tested in all *E. coli* isolates growing on media with colistin by PCR using a set of primers described previously (Table S4). Additionally, direct detection of *mcr* genes in the whole-community DNA isolated from the primary samples enriched in LBB with colistin (1 mg/L) was performed using the same PCR protocols as indicated in Table S4. Results of positive PCR detection of each *mcr* variant in the whole-community DNA were confirmed for the first 200 samples by Sanger sequencing.

### Antimicrobial susceptibility testing

Isolates carrying *mcr* genes were subjected to susceptibility testing to 20 antimicrobial substances by disk diffusion method on Mueller-Hinton agar (Oxoid, UK). The set of antibiotics and their concentrations are listed in the Supplementary Material and Methods: Antimicrobial susceptibility testing. The production of extended-spectrum beta-lactamase (ESBL) and AmpC type beta-lactamase was tested by phenotype diffusion test (D68C1 AmpC & ESBL Detection Set, Mast Diagnostics, UK). In *mcr*-positive isolates, the minimal inhibitory concentration (MIC) to colistin was tested using Mikrolatest MIC plates (Erba Lachema, Czech Republic) and the breakpoint >2 mg/L according to European Committee on Antimicrobial Susceptibility Testing (EUCAST) guidelines (15).

### Whole-genome sequencing and data analysis

This section represents an overview of WGS sequencing and subsequent analysis. All details and references can be found in the Supplementary Material and Methods section. All *mcr*-positive isolates (*n* = 111) were subjected to short-read-based sequencing using Illumina, filtered for quality and assembled with assemblies’ statistics summarized in Table S5. A total of 109 isolates were involved in further analysis as two isolates were excluded due to low data quality. Genomic typing using established tools and databases was used to identify the presence of bacterial sequence types (STs), antibiotic resistance genes (ARGs), plasmid replicons, plasmid sequence types, antibiotic resistance-related chromosomal mutations, virulence-associated genes (VAGs), and phylogenetic groups (PGs) in assemblies. Concordance between antibiotic resistance results observed phenotypically and those assessed from WGS was evaluated. The genetic context of *mcr*-containing region was studied to provide a picture of their probable presence on specific plasmids or being encoded chromosomally. The presence of ColV plasmids was evaluated as they are important markers for *E. coli* virulence. Phylogenetic analysis was performed for 109 *mcr*-positive *E. coli* in our set, and additional phylogenetic analysis was implemented to further study relations of some of the dominant STs, particularly ST1011, or known prominent extraintestinal pathogenic *Escherichia coli* (ExPEC) STs such as ST131 from our set compared to the same STs from other data sets.

### Conjugative transfer of *mcr* genes

The transferability of *mcr* genes into plasmid-free sodium azide-resistant *E. coli* J53 K12 recipient was performed by conjugation assays using filter-mating method (16). Bacterial culture including donor and recipient was cultivated overnight at 37°C on Luria-Bertani agar plates with sodium azide (100 mg/L) and colistin (0.5 mg/L). The successful transfer of *mcr*-carrying plasmid was confirmed by PCR targeting the individual variants of the *mcr* gene and specific PCR for identification of the recipient *E. coli* J53 K12 (17). The approximate size and number of transferred plasmids to recipient cells were determined by pulsed-field gel electrophoresis using S1 nuclease.

## RESULTS

### Selective cultivation and PCR detection

From a total of 659 fresh meat and cecum samples, 320 bacterial isolates were obtained by selective cultivation on two colistin-supplemented agar media (199 isolates from MCA and 121 isolates from EMB agar). Species naturally resistant to colistin (counting 51 isolates) were excluded prior PCR testing, and they are listed in Supplementary Results. A total of 111 *mcr-1*-positive *E. coli* isolates were obtained. No other *mcr* variants or species carrying *mcr* genes were found. The 111 *mcr-1*-positive isolates came from a total of 65 primary samples as two isolates per sample were recovered from 46 samples (one per each media—EMB/MCA). In terms of sample origin, isolates carrying *mcr-1* gene were found in fresh meat from domestic production (43/303, 14.2%) with highest occurrence in poultry (33/149, 22.1%), followed by pork (6/74, 8.1%) and beef (4/80, 5.0%) (*P* < 0.002, *α* = 0.05). Higher levels of *mcr-*carrying isolates were observed from imported meat (17/59, 28.8%) compared to the domestic production, especially from poultry (16/32, 50.0%) and less from beef (1/10, 10.0%). No *mcr*-harboring isolates were found in imported pork samples. The difference in *mcr-1* prevalence between domestic (22.1%) and imported (50.0%) meat was statistically significant only for chicken (*P* < 0.004, *α* = 0.05). In contrast, a low occurrence of *E. coli* with *mcr-1* gene was observed in the intestine of domestic slaughter poultry (3/181, 1.7%) and pigs (2/114, 1.8%). No *mcr*-carrying isolates were found in the cecal samples originating from imported poultry (0/2). The prevalence of *mcr-1* in meat and intestine was significantly different, only in the case of poultry (*P* < 0.00001, *α* = 0.05).

### Direct detection of *mcr* genes in primary samples

**Fig 1:**
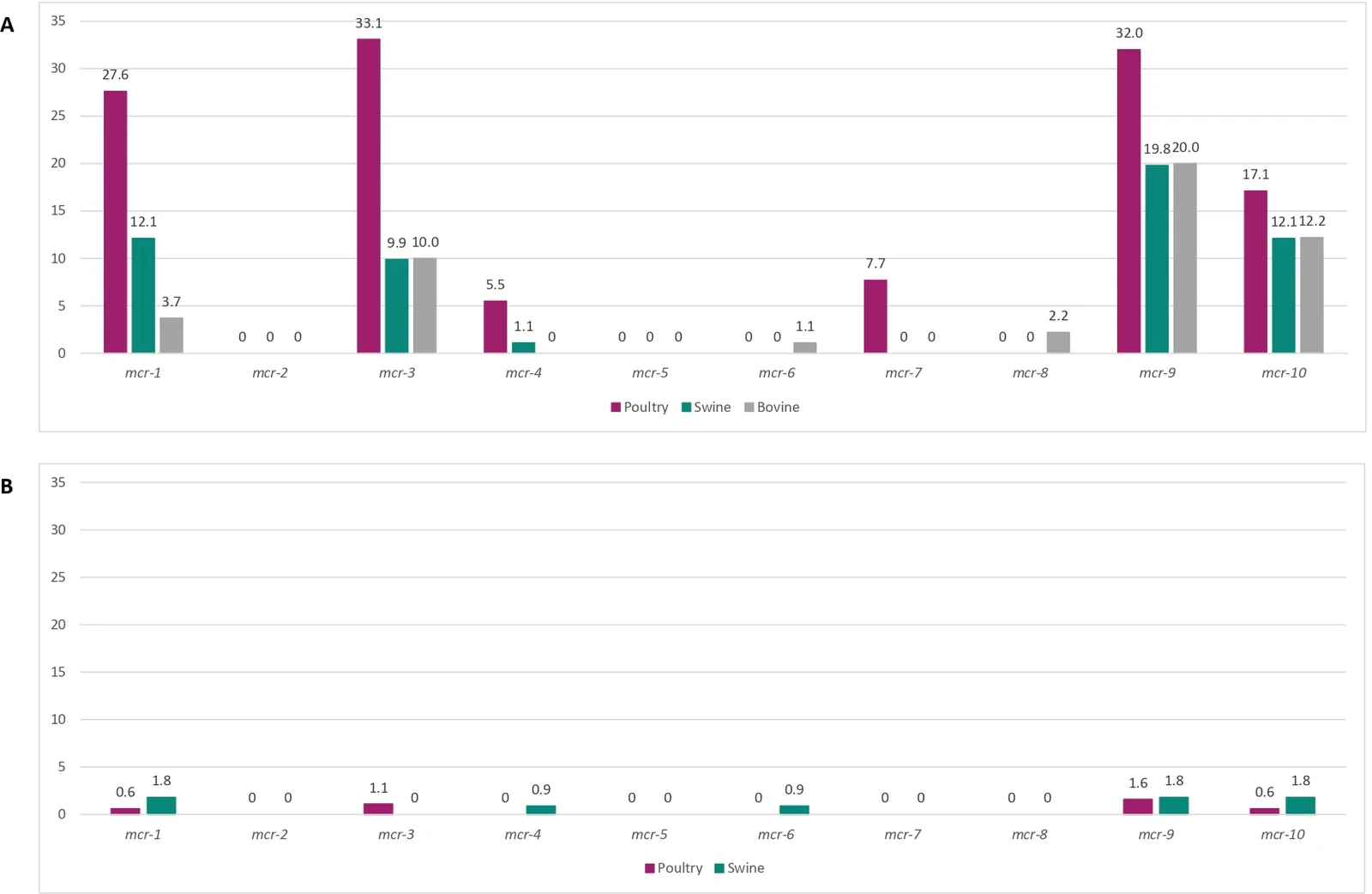
Occurrence (%) of *mcr* genes in total DNA isolated from (A) primary fresh meat and (B) cecal samples enriched in LBB with 1 mg/L colistin. Bovine cecum samples were not available.

PCR detection of *mcr* genes was simultaneously performed in the whole-community DNA isolated from enriched primary samples in LBB with colistin. In contrast to the colistin-resistant *E. coli* isolates obtained by selective cultivation where only *mcr-1* was found, the diversity of *mcr* genes in the whole-community DNA of the primary samples was higher (Fig. 1). At least one variant of *mcr* gene was found in 205 (31.1%) of the primary samples including poultry (62.4%), pork (19.1%), and beef (18.5%). The most common genes included *mcr-9* (99/659, 15.0%), *mcr-3* (80/659, 12.1%), *mcr-1* (70/659, 10.6%), and *mcr-10* (56/659, 8.5%), followed by less common variants *mcr-7* (14/659, 2.1%), *mcr-4* (12/659, 1.8%), and *mcr-8* (2/659, 0.3%). Seventy-three (11.1%) whole-community DNA samples contained multiple *mcr* genes simultaneously, with the most common variant being a combination of *mcr-1* and *mcr-9* (16/659, 2.4%), followed by *mcr-1* with *mcr-3* (15/659, 2.2%) or *mcr-3* along with *mcr-7* (10/659, 1.5%). Thirteen DNA samples (13/659, 2.0%) carried three different variants, namely *mcr-1*, *mcr-3,* and *mcr-9*. The highest occurrence of at least one *mcr* gene variant was observed in poultry meat, both imported (28/32, 87.5%) and domestic (95/149, 63.8%). An increased prevalence of an *mcr* gene was also observed in pork meat from domestic production (27/74, 36.5%) compared to imported pork (4/17, 23.5%). In beef, *mcr* genes were detected in 42.5% (34/80) of domestic meat and in 40.0% (4/10) of meat samples from imports. In the cecal samples from slaughter animals, *mcr* genes were detected in both poultry samples (5/181, 2.8%) and pigs’ samples (8/114, 7.0%) at low level.

### Differences between cultivation on colistin-supplemented media and direct PCR detection of *mcr*

The *mcr-1* gene was the only variant detected in both the whole-community DNA of primary samples (70/659, 10.6%) and on colistin-selective media (65/659, 9.9%). When combining the results of both methods, a total of 79 (11.9%) were found positive for *mcr-1*. From these, 57 samples were tested positive by both cultivation and direct detection approach. Other 22 samples showed inconsistency between the methods when 13 samples were positive only by direct PCR detection, while 8 samples were tested positive only by cultivation.

### Antibiotic susceptibility testing and antibiotic resistance genes detection in *E. coli* isolates

Most *mcr*-positive isolates (97/111, 87.4%) showed an MDR profile defined as resistance to at least one antimicrobial drug in three or more antimicrobial categories.

The most prevalent was resistance to ampicillin (111/111, 100.0%), nalidixic acid (96/111, 86.5%), tetracycline (96/111, 86.5%), streptomycin (93/111, 83.7%), and sulfonamides (90/111, 81.1%). The prevalence of resistance to the other antibiotics can be found in Table S2. Nine isolates were ESBL producers, while 10 isolates were suspected carbapenemase producers or ESBL/AmpC beta-lactamase producers with porin loss.

In terms of genotype of *mcr-1*-positive isolates, ARGs (Table S1) to beta-lactams (104/109, 95.4%), tetracycline (98/109, 89.9%), sulfonamides (86/109, 78.9%), trimethoprim (74/109, 67.9%), and to aminoglycosides (72/109, 66.0%) were found. Two isolates carried ARGs to more than 11 different antibiotics. Moreover, the *mcr*-positive isolates contained ARGs to narrow-spectrum beta-lactams such as *bla*_TEM-1B_ (*n* = 86) and *bla*_TEM-1C_ (*n* = 7). ESBL-positive isolates were found only in fresh poultry meat (9/88, 10.2%) and contained *bla*_CTX-M-1_, *bla*_CTX-M-14b_, *bla*_CTX-M-15_, and *bla*_CTX-M-55_. A total of 16 isolates carried *bla*_TEM-106_ which is supposed to hydrolyze the third-generation cephalosporins according to ResFinder database, but this showed to be problematic as discussed below. Among the genes encoding AmpC beta-lactamases, the *bla*_CMY-2_ gene was detected in one isolate. Chromosomal mutations conferring resistance to quinolones and colistin have been identified. At least 1 of the 10 known mutations in genes *gyrA*, *parC,* and *parE* was detected in 76.1% of isolates (83/109), while 32 isolates carried the transferable mechanisms of quinolone resistance genes. In addition to *mcr* genes, 13 different mutations in *pmrA*/*pmrB* coding for colistin resistance were identified in 66.9% of the isolates (73/109).

### Concordance between phenotypic testing and WGS data of *E. coli* isolates

The phenotype matched genotype for all tested antibiotics in only 38/109 (34.9%) isolates; however, the issues were mainly related to several antibiotics which seemed to be systematically problematic (Table S2). The concordance below 90% was detected for streptomycin (77.5%), cefazolin (85.6%), ceftazidime (83.8%), azithromycin (80.2%), and aztreonam (84.7%).

### Genotypic characterization of *E. coli* isolates

**Fig 2:**
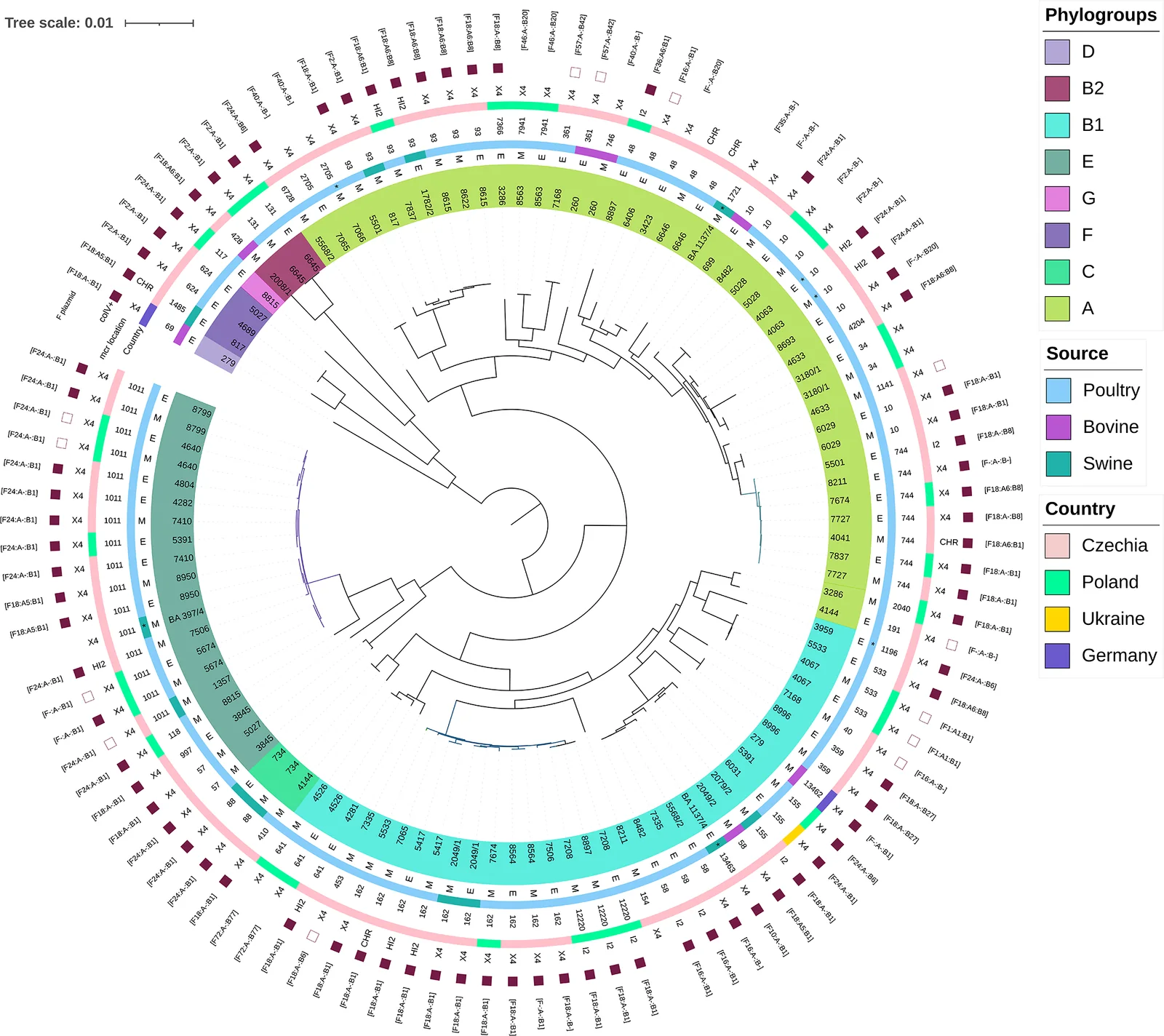
Phylogenetic relationships of 109 *mcr*-positive *E. coli* isolates. The color in the background of the isolate name represents the respective phylogroup (see legend Phylogroups). The first inner metadata circle corresponds to isolation from MCA (M) or EMB (E) and is followed by source column (see legend Source), while the symbol “*” means that the sample was from cecum, not meat. The next columns reveal sequence type, country of origin (see legend Country), and location of *mcr* within specific plasmid or chromosome. The presence of ColV plasmids is highlighted by purple squares, while full square means ColV-positive isolates, and an empty square indicates detection of at least some ColV-related genes from its scheme. The last circle corresponds to the F plasmid type (RST scheme).

Regarding the clonal relationships of *E. coli* isolates (Fig. 2), 109 sequenced *mcr*-positive isolates belonged to 40 different STs and to 8 different PGs with predominance of A (44/109, 41.3%), B1 (34/109, 32.1%), and E (20/109, 19.2%). The most prevalent STs were *E. coli* ST1011 (17/109, 15.6%) from PG E and ST162 (13/109, 12.8%) from PG B1. Both STs (ST1011, ST162) showed almost identical ARG profile, including the chromosomal mutations to quinolones and colistin. Seven isolates were assigned to ST93 and ST744 and one isolate to ST117, which are commonly classified as avian pathogenic *E. coli* (APEC) (18). Among the STs associated with human infections, ST10 (*n* = 9), ST58 (*n* = 5), ST131 (*n* = 2), ST88 (*n* = 1), and ST69 (*n* = 1) were identified. Interestingly, almost half of the samples (23/47, 49%), from which two isolates were obtained, contained isolates of different STs. In case two isolates with the same STs were obtained from the same sample, they commonly represented a clonal pair (up to 10 SNPs). Interestingly, clonal pairs of strains were also observed for isolates coming from different samples. The isolates had various ARGs and VAGs (Fig. S1 and S2) and also commonly (93%) carried F-type plasmids with replicon sequence type (RST) F24:A-:B1 (*n* = 20) and F18:A-:B1 (*n* = 26) being the most prevalent. ColV plasmids were identified in 78/109 (71.6%) of the isolates (Fig. 1; Fig. S3) from all source groups. The *csgA* virulence gene associated with biofilm and increased pathogenicity was detected in all isolates carrying ColV. Moreover, in the majority of *mcr-*positive isolates (100/109, 91.7%), the colonization-facilitating virulence factor *cvaC* was detected.

### Zoonotic *E. coli* ST1011

We compared our data with *mcr-*carrying *E. coli* strains coming from Czech patients sampled during 2010–2020 (19). We observed that several STs overlapped with our study including ST69, ST88, ST131, ST162, ST744, and ST1011 (Table S3). The phylogenetic analysis and SNPs evaluation revealed that strains from patients and meat were distant (minimally hundreds of SNPs) for ST69 (3 isolates), ST88 (5), ST131 (3), and ST162 (12). For ST744 (17), the minimal count in the human-animal/meat pair was 65 SNPs (core genome of 3,888 genes). For ST1011 (20), the minimum count for such a pair was only 38 SNPs (involving 4,073 core genes, with the range 38–3,816 SNPs and average 2,373 SNPs). These numbers do not imply direct transmission of ST1011 between humans and meat samples but indicate a clear zoonotic potential of this ST. Moreover, ST1011 represents the most prevalent sequence type in our study so we decided to investigate its global cohort.

**Fig 3:**
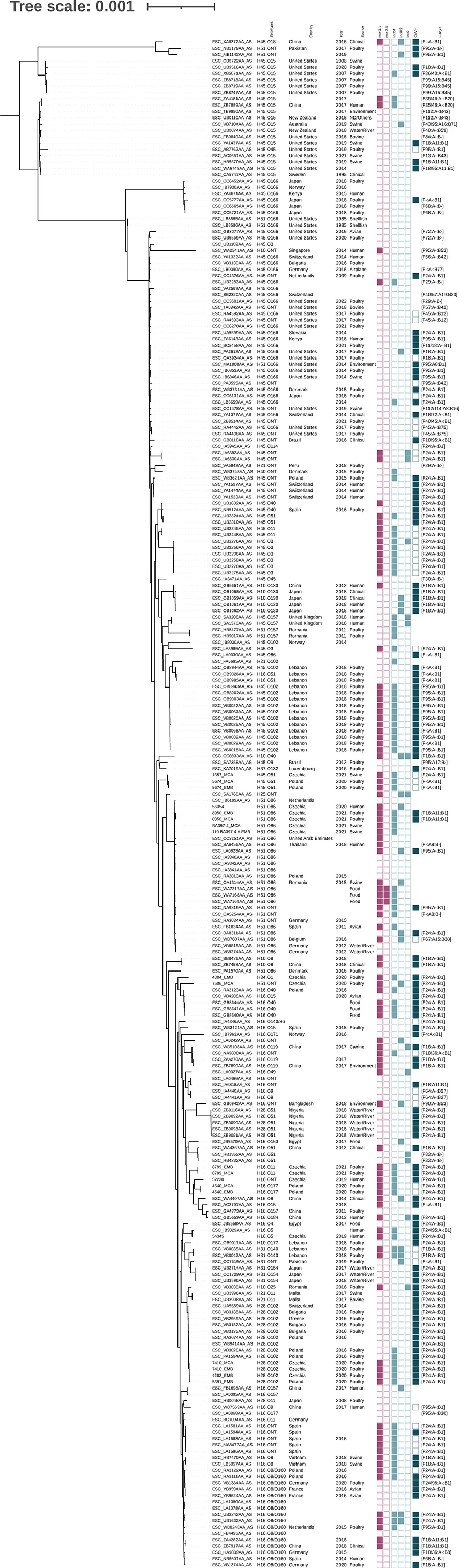
Phylogenetic analysis of the global cohort of ST1011. The isolates with the name starting “ESC” were recovered from EnteroBase (222), while the rest came from our studies (19: 3 humans and 16 animals). The metadata columns reveal serotype, country of origin, year of isolation, source of isolation, presence of mcr-genes (purple squares) and related plasmid replicons (turquoise squares), ColV-positive isolates (see legend Fig 2), and F plasmid type.

According to EnteroBase, the two oldest ST1011 strains were isolated in 1985 and came from a shellfish (US) and the first record of a human strain was from 1995 (Sweden). Overall, the 241 ST1011 isolates (Fig. 3) came from 35 countries and various sources including humans (32 strains, 10 of them clinical), poultry (81), swine (14), water (11), food (8), birds (5, other than poultry), and other (12). For the remaining 79 strains, the source metadata were not available.

We observed that several dominant serotypes can be detected and most of them clustered together (e.g., H45:O15, H10:O130, H51:O86, H45:O102, H28:O102, and H16:O8/O160), while H45:O166 was distributed across two different clades in the phylogenetic tree. We detected 11 different variants of H antigen, while the most prevalent were H45 (41.91%, 101/241), H16 (27.39%, 66/241), and H51 (12.86%, 31/241). For O antigen, there were 33 different types, and O166 (12.86%, 31/241), O102 (11.20%, 27/241), O8 (11.00%, 29/241), and O86 (9.96%, 24/241) were predominant.

The number of VAGs across the ST1011 ranged from 61 to 94 with an average of 72.49 VAGs per strain which are not unusually high numbers for *E. coli* (20, 21). A total of 127 different VAGs were found, and 62 of them were previously associated with Extraintestinal Pathogenic Escherichia coli (ExPEC) pathotype (22 – 24) including the APEC subgroup (Fig. S4). The number of resistance genes was quite various among the ST1011 isolates with an average of 9.33 ARGs per strain. The total of 14 strains carried no ARGs, while 3 strains carried 25 ARGs. Interestingly, the *mcr-1* gene was carried by 41% (99/241) of the strains. If we exclude the 19 strains of ours, given that we selected these strains based on *mcr* presence, *mcr-1* prevalence is still high among the EnteroBase collection with 35.8% (79/222). ColV plasmids were found across the collection in almost half of the strains (49.8%, 120/241) but without a clear phylogeny-specific pattern. We observed the association of several F plasmids with ColV. The most prevalent F plasmid RST, F24:A-:B1, was found in 82 isolates and linked with ColV in 60 cases (73.2%, 60/82). Similarly, ColV-positive strains were associated with F18:A-:B1 (18/20) and F95:A-:B1 (16/18). Plasmids with RST F18:A11:B1 were not prevalent in ST1011 collection (7/241), but interestingly, they were positive for all genes in the ColV scheme.

### Plasmids carrying *mcr* genes in *E. coli* isolates and their transferability

The locations of *mcr-1* were determined using complete plasmid sequences (for three IncH2 plasmids) and estimated using short-read-based assemblies for other isolates. Plasmid alignments suggest that *mcr-1* gene was likely carried on one of three groups of plasmids including IncX4 (88/109, 80.7%), IncI2 (8/109, 7.3%), and IncHI2 (8/109, 7.3%), with five isolates having the *mcr* gene likely incorporated into the chromosome (5/109, 4.6%). Conjugation experiments revealed that *mcr-1* genes were successfully transferred in 83.2% (45/54) of the selected isolates, with the majority belonging to IncX4 (62.9%), followed by IncHI2 (12.9%) and IncI2 (7.4%). The WGS-based estimation of *mcr-1* location was supported by the conjugation experiment results and S1-PFGE size characterization (for the successfully transferred plasmids). The comparative analysis of *mcr* region for isolates from our set, our other studies focusing on *mcr* in Czech Republic (10, 19), and relevant public data obtained from GenBank can be found in Supplementary Results: Genetic context of *mcr*-containing region.

## DISCUSSION

This study investigated the occurrence of *mcr* in fresh meat samples from retails and slaughter animals in the Czech Republic collected between 2020 and 2021. The results showed a significantly higher prevalence of *mcr*-carrying isolates in imported fresh meat (17/59, 28.8%) compared to meat from domestic production (43/303, 14.2%), which may be related to long-term limited usage of colistin in the Czech Republic or specific animal husbandry practices. It has been recently shown that the ban of colistin use as a food additive for animals results in decreased prevalence of *mcr* genes (25). Additionally, the farm biosecurity practices were previously proved to correlate with ARGs prevalence in metagenomes of pigs fecal samples in nine European countries (26).

In cecum samples, only 1.3% isolates carrying *mcr* was detected in poultry and pigs. The low prevalence of *mcr* genes in cecal samples was detected previously (27, 28). Particularly, reference (28) found the prevalence of *mcr-1* in cecal samples to be the lowest compared to fecal samples (highest prevalence) and meat samples at retail (medium prevalence). The differences in microbial communities’ composition across the guts or secondary contamination of the meat are plausible explanations for the higher prevalence of *mcr* in meat samples than in cecal samples; however, other factors may be involved. Therefore, more studies are needed to confirm the lower cecal prevalence of *mcr* as a general trend and to explain the reasons for it.

Meat trade has been recognized as one possible path for the dissemination of *mcr* genes across countries (29). Therefore, the occurrence of plasmid-mediated colistin resistance should be monitored on a regular basis to see its dynamics. The prevalence of *mcr-1* can be highly influenced by colistin usage as observed in China where the ban on colistin use in livestock in May 2017 resulted in significant decrease of *mcr-1* prevalence among pig farms from 45.0% in 2016 to 19.4% in 2018 (25). Interestingly, a recent genome-wide association study aimed to identify the best genomic markers for predicting APEC showed that *udg* gene associated with polymyxin resistance was one of two classifiers that provided the most accurate prediction (22). This suggests that colistin usage to treat colibacillosis in poultry created stable genomic consequences. Similar dependencies may exist in the case of *mcr* genes which may play in favor of *mcr*-positive strains even without further colistin selective pressure. The results of the studies show that poultry are at risk of colistin exposure and may be susceptible to the spread of the *mcr* gene, e.g., via plasmids (14).

In our study, colistin-resistant *E. coli* was identified in 10% of the samples, mainly in chickens and pigs. In comparison, a higher prevalence of plasmid-mediated colistin resistance in meat was also observed in another study from the Czech Republic using the same methodological approach. In that study, 21% of randomly selected retail meat and liver samples of different origins from 13 countries contained isolates of *Enterobacteriaceae* family with *mcr* genes (30). Isolates were obtained mainly from turkey meat from both import and domestic production (67%). In contrast, lower *mcr* prevalence was recorded in France, where the prevalence of *mcr-1* was examined in cecal and fecal samples from pigs (0.5%), broilers (1.8%), and turkeys (5.9%) (31). The lower prevalence compared to our results could be partially explained by the study design, as in the French study no meat samples were included and *mcr* genes were tested in *E. coli* obtained on colistin-free media. The observed differences between studies are often influenced by different methodological approaches which include: (i) the use of colistin-supplemented media for selection of resistant isolates with concentration of colistin ranging from 2 to 4 mg/L (30, 32 – 34), (ii) testing *mcr* genes in all isolates despite their susceptibility profile, or (iii) performing testing in selected isolates based on colistin MIC values. This supports the need to harmonize the surveillance of colistin resistance as we have for ESBL/AmpC/carbapenemases in *E. coli* (35). The recent multicenter evaluation of selective isolation protocol for detection of *mcr*-positive *E. coli* and *Salmonella* spp. (32) represents a great step forward toward this harmonization.

The occurrence of MDR bacteria with resistance to three and more different antibiotics was very common in our collection of *mcr-*positive isolates. Furthermore, the production of ESBL and AmpC beta-lactamases was observed. Plasmids carrying *mcr-1* and ESBL genes simultaneously have been reported in *E. coli* isolates from imported poultry meat in Europe as well as in chickens from Tunisia (36, 37), highlighting the importance of selective pressure of multiple antimicrobial groups in the dissemination of *mcr*.

While we only detected *mcr-1* by the use of selective cultivation of colistin-resistant colonies followed by PCR, direct PCR detection of *mcr* in the whole-community DNA showed higher occurrence of *mcr* genes (31.1%) and presence of various gene variants. The difference in *mcr* prevalence between these two approaches could be mainly caused by the fact that our cultivation protocol targeted only *E. coli* isolates and other species that might have carried *mcr* genes were overlooked. Additionally, the selective concentration of colistin 3.5 mg/L most likely caused that some *E. coli* isolates with *mcr-9* were missed as this gene variant does not provide clinical levels of colistin resistance (38). If we consider the *mcr-1* gene as the only variant detected by both methods, we did not observe any significant differences in the overall prevalence by selective cultivation (9.9%) and direct PCR detection (10.6%). However, quite large portion of samples (22/79) positive for *mcr-1* showed discordance between the methods, suggesting that combining both methods may be the most appropriate approach for the purpose of *mcr* screening. Interestingly, difference in prevalence of *mcr-1* between the two methods was also observed in another study where *mcr-1* was detected in 24.8% (53/214) of retail chicken meat, while the presence of *mcr-1*-positive *Enterobacteriaceae* was confirmed by culture in only 34 from these 53 samples (34/214, 15.9%) (39). *mcr-3* was among the most prevalent variants detected in the whole-community DNA samples in our study, but no isolates carrying this gene were found by cultivation. This gene is associated mainly with *Aeromonas* spp. and has been previously observed across all sample types including environments and humans (40). Moreover, this species is believed to serve as a reservoir of *mcr-3*. Another abundant variant in the whole-community DNA, *mcr-9*, commonly found in *Enterobacter* spp., does not confer phenotypic resistance to colistin (41). Therefore, isolates carrying *mcr-9* cannot be obtained by selective cultivation on colistin-supplemented media. These observations support the importance of combining cultivation methods with direct culture-independent approaches such as PCR detection or metagenomics in order to have a complete picture on distribution and epidemiology of plasmid mediated colistin resistance.

There is an urgent need for rapid and effective detection of antibiotic resistance as culture-based methods are time-consuming. In this regard, genomic data provide a great opportunity to predict the resistance phenotype and have been recently explored by several studies (42, 43). Therefore, beside testing of antibiotic susceptibility by disk diffusion, we used WGS-based genomic data to compare the concordance of phenotype and the genotype of isolates from our collection. Such data will serve as a useful starting point for building machine learning models for predicting antibiotic resistance phenotypes (44). However, quite high discordance, especially for some antibiotic groups such as aminoglycosides, macrolides but also beta-lactams, has been observed in our study and also in previous reports (42, 43). The binary scheme of genetic marker presence/absence might not always reflect the phenotype precisely for various reasons such as different levels of gene expression related to genetic context (e.g., presence of adjacent promotor). Therefore, more effort is needed before WGS-based resistance prediction can be reliably implemented in clinical practice for selecting optimal antibiotic treatment. The common cause of discordance between genotype and phenotype for streptomycin and azithromycin, respectively, was related to the detection of no genetic marker (GM, Table S2)." ( which would correspond with observed resistant phenotype. We are, therefore, unable to identify the specific background for this discordance, but it suggests our knowledge in this field as well as our databases is still limited. We may miss some markers of resistance, or there is an interplay of multiple factors which could be hard to uncover from genomic data. On the other hand, the problem with beta-lactam antibiotics (cefazolin, ceftazidime, and aztreonam) was largely related to the presence of GM which is expected to provide resistance to the specific antibiotics, but this expectation has not always been fulfilled. This issue was very often connected with the presence of *bla*_TEM-106_ which should have the ability to hydrolyze higher generation of beta-lactams according to the ResFinder database so the function of this gene should be revalidated.

The phylogenetic analysis revealed high genetic diversity of *mcr*-positive isolates including the presence of internationally recognized high-risk clones such as ST10, ST58, ST117, ST93, ST162, ST131, ST744, and ST1196 (45, 46). Importantly, ST10, ST58, ST69, ST88, ST117, ST131, and ST410 detected within this study also belong to the 20 most prominent ExPEC STs responsible for over 85% of ExPEC-related infections globally (47). Multiple isolates of *mcr-1*-positive *E. coli* of ST93, ST744, and ST1011 were identified in poultry in Lebanon (48) suggesting these STs might be associated commonly with colistin resistance and avian hosts.

*E. coli* ST1011 and ST162 are globally distributed and have been found in various sources (49, 50). Several *mcr-1*-associated STs including ST744, ST10, and ST162 also alarmingly overlap between livestock and clinical settings (51, 52). We observed this phenomenon while comparing the data from our study and another project of our team that was focused on human patients in the Czech Republic (19). Despite no detection of a clonal pair of isolates with meat and human origin, we detected closely related isolates with a minimum of 38 SNPs in case of ST1011. Given the phylogenetic analysis in this particular case involved a remarkably high number of core genes (4,073), the observed difference between human and animal isolates of ST1011 is indeed very low in this context. It was previously suggested that *E. coli* STs with lower pathogenic but high zoonotic potential such as ST10 and ST155 represent a crucial reservoir of *mcr-1* due to their ability of acquiring ARGs and their high prevalence in animals and humans (53).

ST1011 has been recognized as MDR in multiple studies in countries including Germany [manure sample (54)], China [human, associated with NMD-9 (55)], Egypt [clinical (49)], [chicken and beef (56)], and Great Britain [pigs (57)]. Here we brought the first data on the global phylogeny of this ST and demonstrated its association with plasmid-mediated colistin resistance. Our analysis of EnteroBase data revealed its distribution even more broadly across different sources and countries and showed that the prevalence of *mcr-1* can be even higher compared to other *mcr-1*-associated lineages such as ST10, ST117, ST156, ST744, and ST457 (52, 58). Moreover, we showed that ST1011 may carry an extensive number of ARGs (up to 25). While the quantity of VAGs was not unusually high among ST1011 comparing to other *E. coli* STs (20, 59), we commonly detected APEC-associated VAGs (24, 60, 61). We demonstrated that ST1011 is frequently linked with the presence of ColV plasmids. ColV plasmids are well known for carriage of typical APEC virulence factors and are considered as one of the main APEC markers (62, 63). ColV plasmids were previously associated with important human ExPEC lineages including ST95, ST117, and sub-lineage of ST58 which are also typically found in poultry (63). Therefore, ST1011 as a globally dispersed zoonotic APEC-associated lineage with the potential to cause human infections deserves to be monitored further.

Plasmid-mediated colistin resistance is primarily associated with conjugative plasmids. More than 10 plasmid incompatibility groups have been described to carry *mcr-1* (53, 64). However, the globally dominant are IncX4, IncHI2, and IncI2 which together count for more than 90% of plasmid types carrying this gene (53, 64). Interestingly, IncX4 was firstly the least abundant with IncHI2 dominance in Europe and IncI2 in Asia, but nowadays IncX4 has become dominant (53, 64, 65). The results of our study correspond to the previous observations as *mcr-1* was likely located mainly on IncX4 replicons, but also on IncI2 and IncHI2 plasmids. These plasmid types harboring *mcr-1* have been found in both animal and human isolates before (10, 53, 66). We observed the highest success rate of conjugative transfer for IncX4 plasmids. Although this observation is limited to our laboratory conditions, it may partially explain the increasing dominance of IncX4 in *mcr-1* spread. The high efficiency of horizontal transfer via this plasmid family poses a significant risk as colistin is considered a last-line antibiotic for the treatment of life-threatening infections in humans.

Our study confirmed the importance of livestock as a major reservoir of plasmid-mediated colistin resistance and pointed out the risks of meat treat for the transmission of *mcr* genes toward humans and between regions with various colistin consumption. We identified several *mcr*-associated prevalent STs, especially ST1011, which should be monitored further as they represent zoonotic bacteria circulating between different environments. Our results also showed the importance of combining cultivation with PCR-based testing of the primary samples. Surveillance of plasmid-mediated colistin resistance is essential not only to prevent its spread among animals but also to adapt treatment strategies accordingly, thereby preventing the development of difficult-to-treat infections that pose a risk primarily to public health.

## Data Availability

The genome assemblies are available under BioProject accession number PRJNA907011, biosample numbers SAMN31968641–SAMN31968751 (Table S1), and complete sequences of three IncHI2 plasmids under accession numbers OQ078085 (p1782/2_EMB), OQ078086 (p4281_EMB), and OQ078087 (p5417_MCA).

## SUPPLEMENTAL MATERIAL

Supplemental file 1 spectrum.00609-23-s0001.pdf Supplemental figures and tables.

ASM does not own the copyrights to Supplemental Material that may be linked to, or accessed through, an article. The authors have granted ASM a non-exclusive, world-wide license to publish the Supplemental Material files. Please contact the corresponding author directly for reuse.
