# Supplementary material for: Plasmid-mediated colistin resistance from fresh meat and slaughtered animals in the Czech Republic: nation-wide surveillance 2020–2021
Source: Microbiol Spectr. 2023 Sep 12;11(5):e00609-23. doi: 10.1128/spectrum.00609-23 (PMC10580956; doi:10.1128/spectrum.00609-23)
Supplement: Supplemental file 1 — Supplemental figures and tables. [file spectrum.00609-23-s0001.pdf]

Supplementary Material and Methods

Sampling and selective cultivation

Samples were taken from different parts of the country according to the methodological procedure by the State Veterinary Institute in Prague as part of the harmonised monitoring of antimicrobial resistance in zoonotic and indicator bacteria (1, 2). The veterinary inspector took at least 300 g of chilled meat(preferably packaged) from the retail outlet, irrespective of the country of origin. In the case of appendix, samples were taken randomly from animals at slaughter after evisceration. On the day of slaughter, ten caecum samples were taken from ten randomly selected carcasses from the same batch and the same epidemiological unit (using a sterile scalpel and tweezers).

Antimicrobial susceptibility testing

The set of antibiotics included: amoxicillin/clavulanate (20/10 µg), ampicillin (10 µg), azithromycin (15 µg), aztreonam (30 µg), cefazolin (30 µg), ceftazidime (30 µg), ciprofloxacin (5 µg), ertapenem (10 µg), fosfomycin (200 µg), gentamicin (10 µg), chloramphenicol (30 µg), imipenem (10 µg), meropenem (10 µg), nalidixic acid (30 µg), nitrofurantoin (300 µg), streptomycin (10 µg), sulphonamides (300 µg), tetracycline (30 µg), tigecycline (15 µg) and trimethoprim/sulfamethoxazole (1.25/23.75 µg). Data were evaluated according to Clinical and Laboratory Standards Institute (CLSI) guidelines (3).

Whole-genome sequencing and data assembly

All *mcr*-positive isolates were subjected to WGS. Genomic DNA of the isolates was obtained using the NucleoSpin Tissue kit (Macherey-Nagel, Germany). DNA libraries were prepared using the Nextera XT DNA Library Preparation Kit (Illumina, San Diego, CA, USA)

and sequenced on NovaSeq 6000 (Illumina, San Diego, CA, USA). The sequencing data of poor quality (Q≤20) and sequencing adapters were removed by Trimmomatic v0.36. (4) and then assembled using SPAdes v3.13.1. software (5). Whole-genome assemblies of two *mcr*-positive isolates (6407_MCA and 6407_EMB) were excluded from further analyses due to low data quality.

Data analysis - ST, antibiotic resistance, plasmid replicons and virulence factors

Bacterial sequence types (MLST 2.0), antibiotic resistance genes (ARGs) (ResFinder 4.1), plasmid replicons (PlasmidFinder 2.1) and plasmid sequence types (pMLST 2.0) were determined using CGE tools available at http://www.genomicepidemiology.org. To detect chromosomal mutations responsible for resistance to quinolones (*gyr/par*) and colistin (*pmrA/pmrB*), PointFinder was used. The virulence-associated genes (VAGs) were detected using the Virulence Factor Database (6). Unless specified differently, thresholds were set at minimum level of 95% coverage of the query sequence and 95% identityfor detection of ARGs and 90% coverage of the query sequence and 90% identity for VAGs and plasmid replicons. The phylogenetic group (PG) of *E. coli* isolates was determined by ClermonTyping (7).

Concordance between antibiotic resistance results observed phenotypically and assessed from WGS

The resistance phenotype results were compared with data obtained from WGS. To assess the expected phenotype, ResFinder 4.1 and PointFinder databases was used. We checkedthe presence of a genetic marker (GM) responsible for any observed resistance to each evaluatedantibiotic. We also checked if no relevant GM was present when we observed a susceptible phenotype. If no expected GM was detected for beta-lactams and quinolones, we

also screened for additional chromosomal mutations within relevant genes that may be responsible for the observed. For beta-lactams (except carbapenems), we accepted any kind of beta-lactamase encoding gene or chromosomal mutations in *ampC* as sufficient to explain resistance to antibiotics from this group. Two antibiotics, colistin and nitrofurantoin, were not involved in this analysis. Colistin was not included because both phenotypic resistance and presence of *mcr*-genes were selective criteria for all isolates in the analysis. For nitrofurantoin, the WGS- based resistance database was not embedded in ResFinder at the time of use.

Genetic context of *mcr*-containing region

To determine genetic context of *mcr* gene in our collection, assemblies were analysedusing BLAST algorithm (https://www.ncbi.nlm.nih.gov/BLAST) in combination with Geneious 9.1.8 software and the ISfinder database (8). Circular comparison ofplasmid sequences with highly similar complete plasmids from the GenBank database was performed using BRIG v0.95 (9).

Of eight isolates harbouring *mcr-1* on a IncHI2 plasmid, three representatives were selected based on the above analysis for long-read sequencing on MinION platform to obtain complete plasmid sequences (Oxford Nanopore Technologies, ONT, Oxford, UK). Genomic DNA was extracted using Genfind V3 (Beckman Coulter, USA), libraries were constructed using an SQK-RBK004 rapid barcoding 1D kit and sequenced on a flow cell (FLO-MIN106 R9.4 SpotON) for 48 hours. All steps were performed according to the manufacturer's instructions. The raw fast5 electrical signals were base called using Guppy v4.2.2 (ONT) to obtain raw reads in fasta format. The raw data were adapter and quality ($Q \leq 9$) trimmed usingBBDuk (https://jgi.doe.gov/data-and-tools/software-tools/bbtools/bb-tools-user-guide/) followed by demultiplexing using Porechop v0.2.4 (ONT). Complete plasmid

sequences wereassembled using Unicycler v0.4.8 (10) and Flye v2.6 (11). Assemblies were polished by Racon v1.4.20 (12) and Medaka v1.2.3 (13) usinglong reads and by Pilon v1.23 (14) using short reads.

ColV plasmids

The presence of ColV plasmids was evaluated using a predefined scheme (15). This scheme considers an isolate ColV-positive if at least one gene is present in minimally four of six defined gene sets: 1) *cvaABC* and *cvi* (the ColV operon), 2) *iroBCDEN*, 3) *iucABCD* and *iutA*, 4) *etsABC*, 5) *ompT* and *hlyF*, and 6) *sitABCD*. We applied the same thresholds for identity (90.0%) and coverage (95.0%) as defined by (15) to screen for the genes using ABRicate (16).

Phylogenetic analysis

The phylogenetic analysis of our 109 strains was performed using Prokka open readingframes prediction (16) and multi-fasta alignment using Roary v.3.12.0 (17). The tree was generated using RAxML (18) supported by 100 bootstraps.The phylogenetic relatedness of the isolates was processed by analysing single nucleotide polymorphisms (SNPs) evaluated using snp-dists (https://github.com/tseemann/snp-dists). The phylogenetic tree together with additional datasets were visualised using the Interactive Tree Of Life (iTOL) (19). We observed that some STs detected in our study were present also in our parallel study focusing on colistin-resistant *E. coli* from humans (20). Particularly, these were ST69 (two human strains), ST88 (n=2), ST131 (n=1), ST162 (n=2), ST744 (n=10) and ST1011 (n=3). We performed additional phylogenetic analysis using the same approaches for each ST separately to compare the strains of human origin withthe strains of animal/meat origin presented here. Based on the analysis, we decided to evaluate ST1011 more deeply.

Therefore, 222 strains from EnteroBase accessed on 2nd April 2022 were downloaded and the phylogenetic analysis of the global cohort (241 strains) was performed,including also 19 of our strains (16 from meat and 3 of human origin).

Supplementary Results

Selective cultivation and PCR detection: Naturally colistin-resistant isolates

Naturally colistin-resistant species excluded from further screening for *mcr* genes by PCR were represent by a total of 51 isolates of *Morganella morganii* (n=20), *Serratia* spp. (n=15),*Moellerella wisconsensis* (n=11), *Providencia alcalifaciens* (n=3) and *Proteus mirabilis* (n=2).

Comparative analysis of *mcr*-region

The shot-gun sequencing did not resolve plasmid sequences in majority of IncX4 plasmids and contigs with *mcr-1* gene varied in size (5,745 bp - 33,568 bp). In 84 isolates, the *mcr-1* genetic context was 100% identical and was characteristic by *parA* and two ORFs encoding a hypothetical protein upstream of the *mcr-1* gene and a gene for PAP2 transmembrane protein downstream of *mcr-1*. Only the isolate 4144_EMB showed different surroundings, the *mcr-1* was bordered by a transposase gene for IS*5* family IS*Kpn26*-like element downstream and a gene encoding PAP2 protein upstream. In three remaining isolates,it was not possible to assess the *mcr-1* surroundings from the obtained WGS data. The described*mcr-1* genetic context has been observed in our previous studies in *E. coli* and *Klebsiella pneumoniae* isolates originating from patients and raw meat of domestic production in the Czech Republic (20, 21) and meat imported to the Czech Republic from Brazil, Germany, and Poland (21). The circular comparison of plasmids from the study and

above mentioned collections showed high similarity (Figure S5). However, we cannot conclude if our plasmids were identical since this analysis was limitedby usage of short-read data.

The contigs with *mcr-1* gene on IncI2 plasmids varied in size (19,469 bp - 60,907 bp) and *mcr-1* gene was localised downstream *nikB* gene as was observed previously (21). Based on shot-gun sequencing data, five out of eight IncI2 plasmids showed high identity (≥ 98% coverage and similarity) to IncI2 plasmid pDR164 (GenBank n. MK542639.1)originating from *E. coli* ST2280 from a black kite in Russia (Figure S6a) (22).The three other IncI2 plasmids showed high identity (≥ 99% coverage and similarity) to many IncI2 plasmids in the GenBank database including human and animal isolates of several species (Figure S6b). This second group of our IncI2 plasmids were also highly similar (≥ 99% coverage and similarity) to IncI2 plasmids previously reported in the Czech Republic (Figure S6b) originating from *E. coli* ST162 and *Citrobacter braakii*, both isolated from imported rabbit meat (China) (21), and from a clinical isolate of *E. coli* ST538 (20).

IncHI2 plasmids carrying *mcr-1* (n=8) belonged to ST4 and were derived from *E. coli* of different STs (ST10 n=2, ST93 n=2, ST162 n=2, ST641 and ST1011). Three IncH2 plasmidssubjected to MinION sequencing showed a plasmid backbone composed of regions for plasmid maintenance, replication, and conjugation transfer but varied in size and composition ofresistance genes. Gene *mcr-1* was flanked by a transposase gene for IS*30* family IS*ApI1*-like element upstream and a gene encoding PAP2 protein downstream in plasmids p1782/2_EMB (291,5 kb) and p4281_EMB (230,6 kb). In p5417_MCA (198 kb), it was surrounded by a transposase gene for IS*1* family IS*1A*-like element upstream and a gene encoding PAP2 proteindownstream. In addition to *mcr-1* gene, p1752/2_EMB carried a broad range of ARGs encoding resistance to aminoglycosides, beta-lactams, macrolides, lincosamides, fluoroquinolones, trimethoprim and sulfonamides while p4281_EMB carried

gene encoding resistance to aminoglycosides, lincosamides and fluoroquinolones and p5417_MCA only to aminoglycosides. Genes conferring tellurium resistance (*terABCDEFWYZ*) were detected in allthree plasmids while p1782/2_EMB also carried genes conferring resistance to copper (*pcoABCDERS* and *cusF*) and silver (*silABCEPRS*). BlastN analysis did not reveal any highly identical IncHI2 plasmids, therefore a circular comparison included only three IncHI2 plasmidsfrom our study and seven other complete IncHI2/ST4 plasmids carrying *mcr-1* and originatingfrom the Czech Republic (20, 21) (Figure S7).

In five isolates, the *mcr-1* gene was carried chromosomally. Contigs harbouring *mcr-1* varied in size (13,571 bp – 182,547 bp) but the genetic surroundings of *mcr-1* were identical. Itwas flanked by a ΔIS*66* (131 bp), followed by a gene encoding PAP2 protein downstream of the *mcr-1* gene and two parts (222 bp and 156 bp) of ΔIS*66* followed by *tnpB* gene upstream of*mcr-1* gene. Inside of these two parts of ΔIS*66*, a 247 bp part of ΔIS*4* was inserted. Also ΔIS*110* (423 bp) and a methyltransferase-encoding gene were localised downstream of this *mcr-1* cassette, and genes encoding a hypothetical protein and GTPase family protein were detected upstream. Similar genetic composition was documented previously in *E. coli* isolates obtainedfrom turkey nd pigs in Italy in 2014 and 2015, respectively (23).

Figure S1: Phylogenetic analysis of the *E. coli* isolates involved in this study with emphasis on antibiotic resistance and plasmid-related metadata.

The metadata in columns reveals media of isolation (Media); sequence type (ST); phylogenetic group (PG); source of origin (Source, see Figure 1 legend); year of isolation (Year); country of origin (Country, see Figure 1 legend); locality of origin (Locality): Vsetin (VS), Pardubice (PAR), Frýdek-Místek (FM), Usti nad Orlici (US), Strakonice (STR), Jicin (JI), Semily (SE), Jindrichuv Hradec (JH), Jablonec nad Nisou (JN), Kolin (KO), Sumperk (SUM), Benesov (BE), Brno-venkov (BRN), Bruntal (BRU), Nachod (NA), Prerov (PRE), Havlickuv Brod (HB), Karvina (KA), Jesenik (JES), Mlada Boleslav (MB), Olomouc (OL), Beroun (BER), Kromeriz (KR)Semily (SE), Rychnov nad Kneznou (RK), Novy Jicin (NJ), Teplice (TE), Trebic (TR), Melnik (M), Zdar nad Sazavou (ZS), Prostejov (PRO), Opava (OP), Prague (PRH), Uherske Hradiste (UH), Ostrava-mesto (OS), Ceska Lipa (CJ); *mcr-1* gene variant (full magenta square means presence); location of *mcr* within specific plasmid or chromosomally (CHM) (mcr on); success of conjugation transfer of *mcr-1* gene (Conjugation transfer).

The presence of respective antibiotic resistance genes besides *mcr* is highlighted by pink squares while blue squares represent presence of respective chromosomal mutation related to resistance to quinolones and colistin. The following column reveals minimal inhibitory concentration for colistin (MIC (Colistin)). The purple circles represent antibiotic resistance phenotypes while full circle means resistance, empty circle intermediate phenotype and no circle represent susceptibility to respective antibiotics. The green squares represent the presence of plasmid replicons (full square). The last column specifies the RST formula for F plasmids.

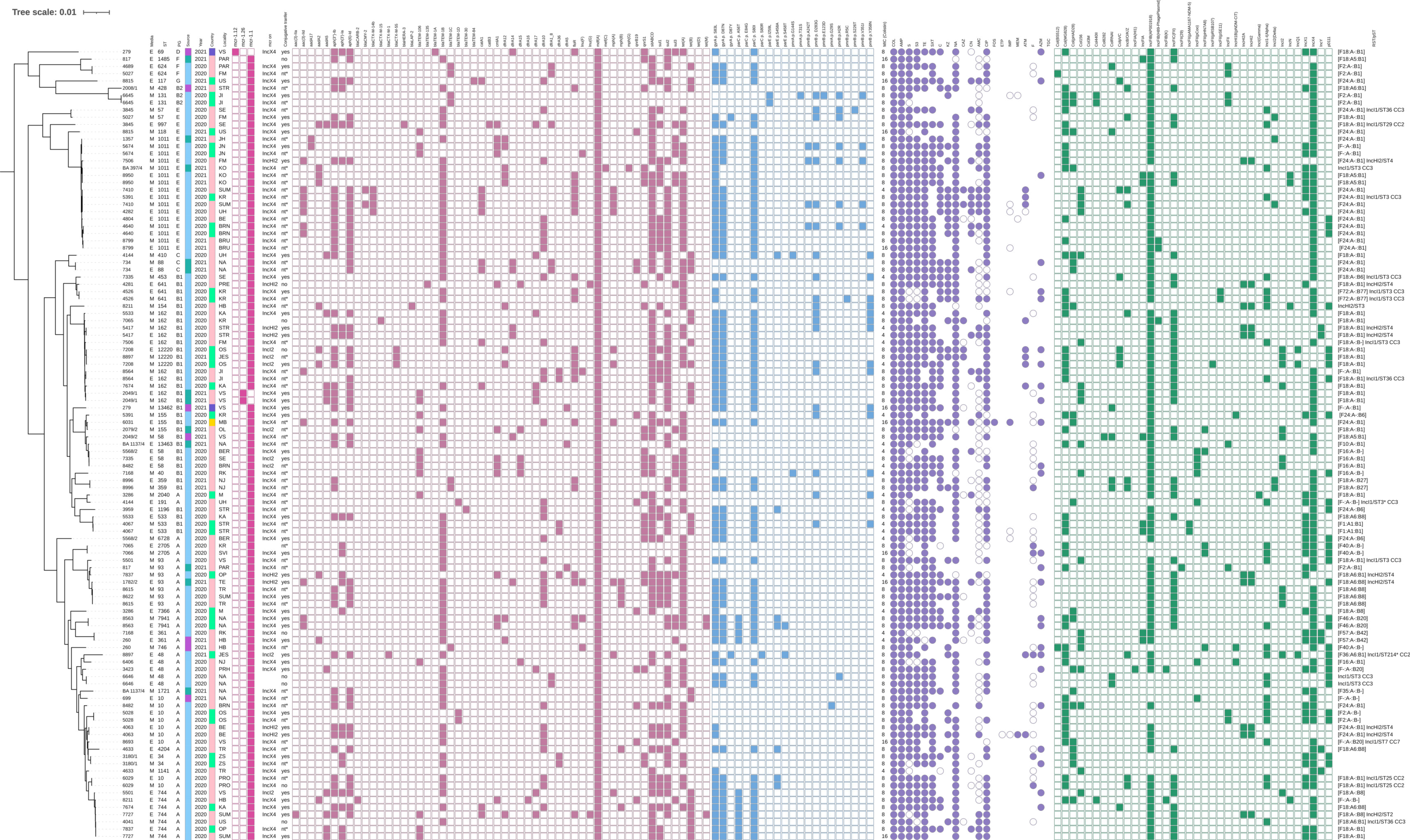
Tree scale: 0.01
Media
ST
PG
Source
Year
Country
Locality
mcr-1.12
mcr-1.26
mcr-1.1
mcr on
Conjugative transfer
MIC (Colistin)
RST/pST

Figure S2: Phylogenetic analysis of the *E. coli* isolates involved in this study with emphasis on virulence-associated genes.

The first seven columns of metadata correspond to **Figure S1**. The magenta squares (full) indicate presence of respective VAGs.

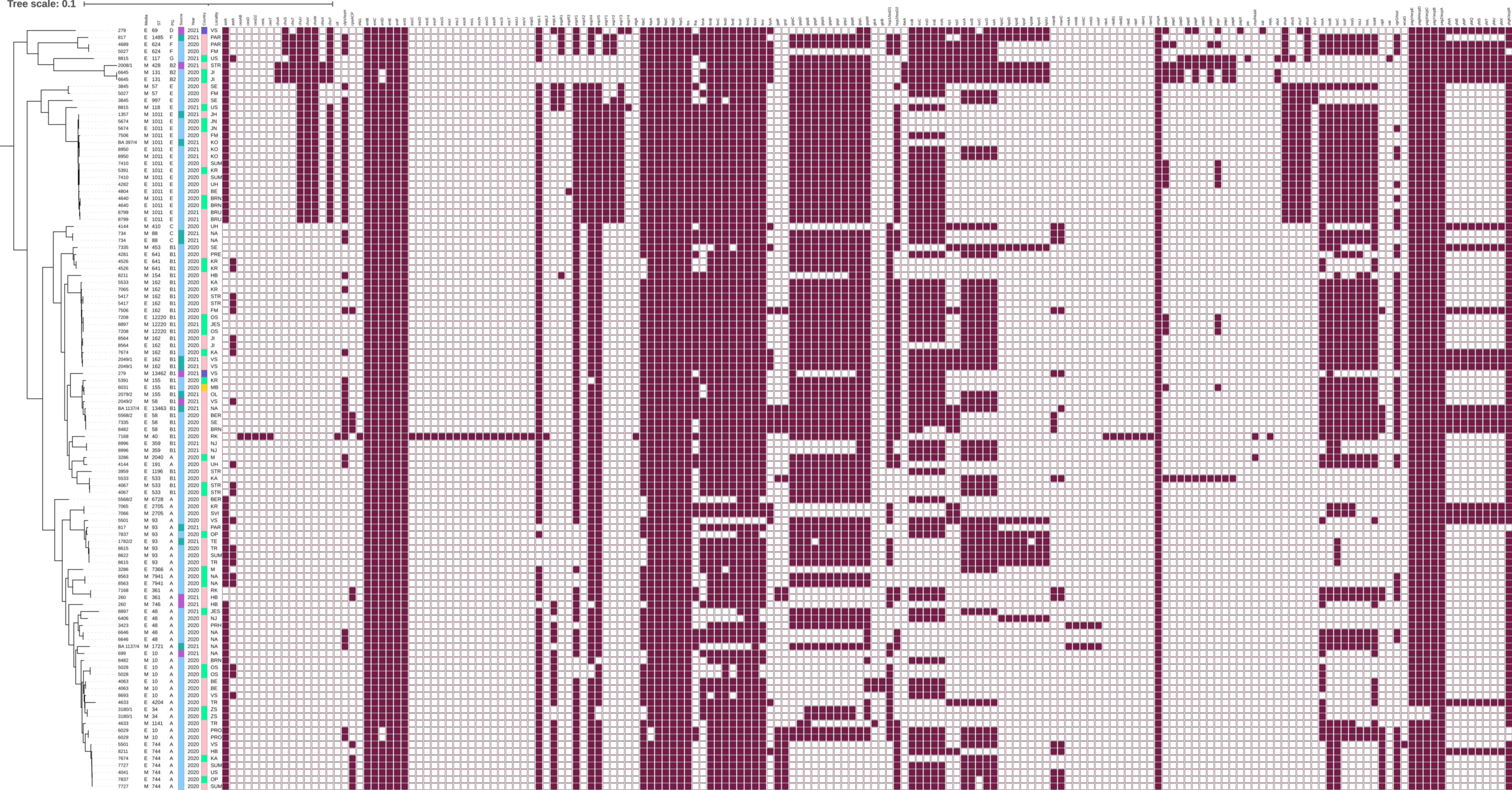
Tree scale: 0.1

Figure S3: Phylogenetic analysis of the *E. coli* isolates involved in this study with emphasis on ColV plasmid markers.

The first seven columns of metadata correspond to **Figure S1**. The darker violet column (full square) indicate ColV-positive strain while lighter columns reveal presence of specific markers involved in the ColV decision scheme.

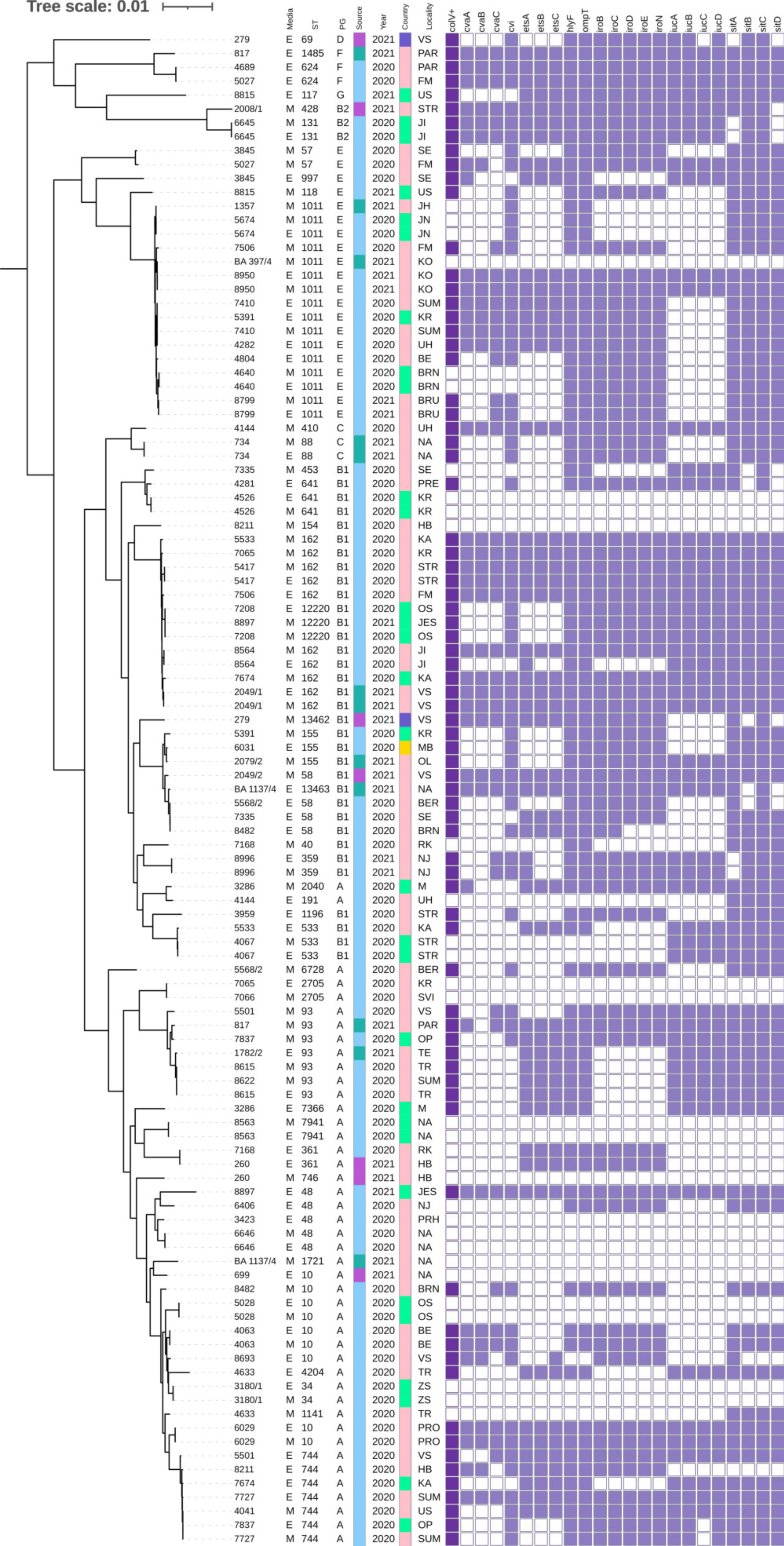
Tree scale: 0.01
Media
ST
PG
Source
Year
Country
Locality
colV+
cvaA
cvaB
cvaC
cvi
etsA
etsB
etsC
hlyF
ompT
iroB
iroC
iroD
iroE
iroN
iucA
iucB
iucC
iucD
sitA
sitB
sitC
sitD
279 E 69 D 2021 VS
817 E 1485 F 2021 PAR
4689 E 624 F 2020 PAR
5027 E 624 F 2020 FM
8815 E 117 G 2021 US
2008/1 M 428 B2 2021 STR
6645 M 131 B2 2020 JI
6645 E 131 B2 2020 JI
3845 M 57 E 2020 SE
5027 M 57 E 2020 FM
3845 E 997 E 2020 SE
8815 M 118 E 2021 US
1357 M 1011 E 2021 JH
5674 M 1011 E 2020 JN
5674 E 1011 E 2020 JN
7506 M 1011 E 2020 FM
BA 397/4 M 1011 E 2021 KO
8950 E 1011 E 2021 KO
8950 M 1011 E 2021 KO
7410 E 1011 E 2020 SUM
5391 E 1011 E 2020 KR
7410 M 1011 E 2020 SUM
4282 E 1011 E 2020 UH
4804 E 1011 E 2020 BE
4640 M 1011 E 2020 BRN
4640 E 1011 E 2020 BRN
8799 M 1011 E 2021 BRU
8799 E 1011 E 2021 BRU
4144 M 410 C 2020 UH
734 M 88 C 2021 NA
734 E 88 C 2021 NA
7335 M 453 B1 2020 SE
4281 E 641 B1 2020 PRE
4526 E 641 B1 2020 KR
4526 M 641 B1 2020 KR
8211 M 154 B1 2020 HB
5533 M 162 B1 2020 KA
7065 M 162 B1 2020 KR
5417 M 162 B1 2020 STR
5417 E 162 B1 2020 STR
7506 E 162 B1 2020 FM
7208 E 12220 B1 2020 OS
8897 M 12220 B1 2021 JES
7208 M 12220 B1 2020 OS
8564 M 162 B1 2020 JI
8564 E 162 B1 2020 JI
7674 M 162 B1 2020 KA
2049/1 E 162 B1 2021 VS
2049/1 M 162 B1 2021 VS
279 M 13462 B1 2021 VS
5391 M 155 B1 2020 KR
6031 E 155 B1 2020 MB
2079/2 M 155 B1 2021 OL
2049/2 M 58 B1 2021 VS
BA 1137/4 E 13463 B1 2021 NA
5568/2 E 58 B1 2020 BER
7335 E 58 B1 2020 SE
8482 E 58 B1 2020 BRN
7168 M 40 B1 2020 RK
8996 E 359 B1 2021 NJ
8996 M 359 B1 2021 NJ
3286 M 2040 A 2020 M
4144 E 191 A 2020 UH
3959 E 1196 B1 2020 STR
5533 E 533 B1 2020 KA
4067 M 533 B1 2020 STR
4067 E 533 B1 2020 STR
5568/2 M 6728 A 2020 BER
7065 E 2705 A 2020 KR
7066 M 2705 A 2020 SVI
5501 M 93 A 2020 VS
817 M 93 A 2021 PAR
7837 M 93 A 2020 OP
1782/2 E 93 A 2021 TE
8615 M 93 A 2020 TR
8622 M 93 A 2020 SUM
8615 E 93 A 2020 TR
3286 E 7366 A 2020 M
8563 M 7941 A 2020 NA
8563 E 7941 A 2020 NA
7168 E 361 A 2020 RK
260 E 361 A 2021 HB
260 M 746 A 2021 HB
8897 E 48 A 2021 JES
6406 E 48 A 2020 NJ
3423 E 48 A 2020 PRH
6646 M 48 A 2020 NA
6646 E 48 A 2020 NA
BA 1137/4 M 1721 A 2021 NA
699 E 10 A 2021 NA
8482 M 10 A 2020 BRN
5028 E 10 A 2020 OS
5028 M 10 A 2020 OS
4063 E 10 A 2020 BE
4063 M 10 A 2020 BE
8693 E 10 A 2020 VS
4633 E 4204 A 2020 TR
3180/1 E 34 A 2020 ZS
3180/1 M 34 A 2020 ZS
4633 M 1141 A 2020 TR
6029 E 10 A 2020 PRO
6029 M 10 A 2020 PRO
5501 E 744 A 2020 VS
8211 E 744 A 2020 HB
7674 E 744 A 2020 KA
7727 E 744 A 2020 SUM
4041 M 744 A 2020 US
7837 E 744 A 2020 OP
7727 M 744 A 2020 SUM

Figure S4: Phylogenetic analysis of the global cohort of ST1011 with emphasis on antibiotic resistance markers, ColV plasmids and APEC-related VAGs.

The first four metadata columns correspond to **Figure 3**. The purple squares area indicates presence of ARGs (full square), the turquoise squares area indicates ColV+ strains and show which markers were present (full) while strains with no markers have no squares; the green area represents presence of APEC-related VAGs.

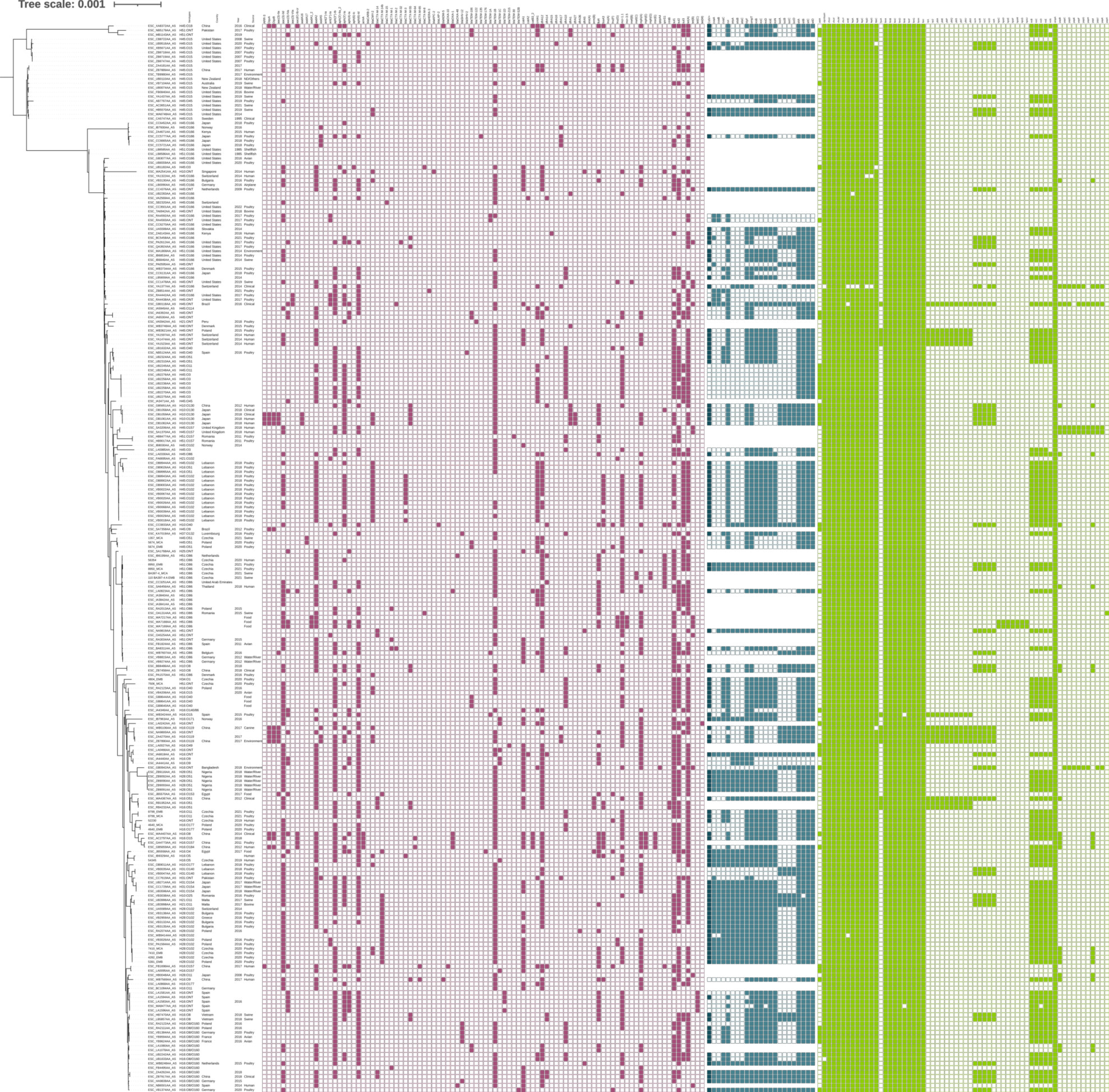
Tree scale: 0.001

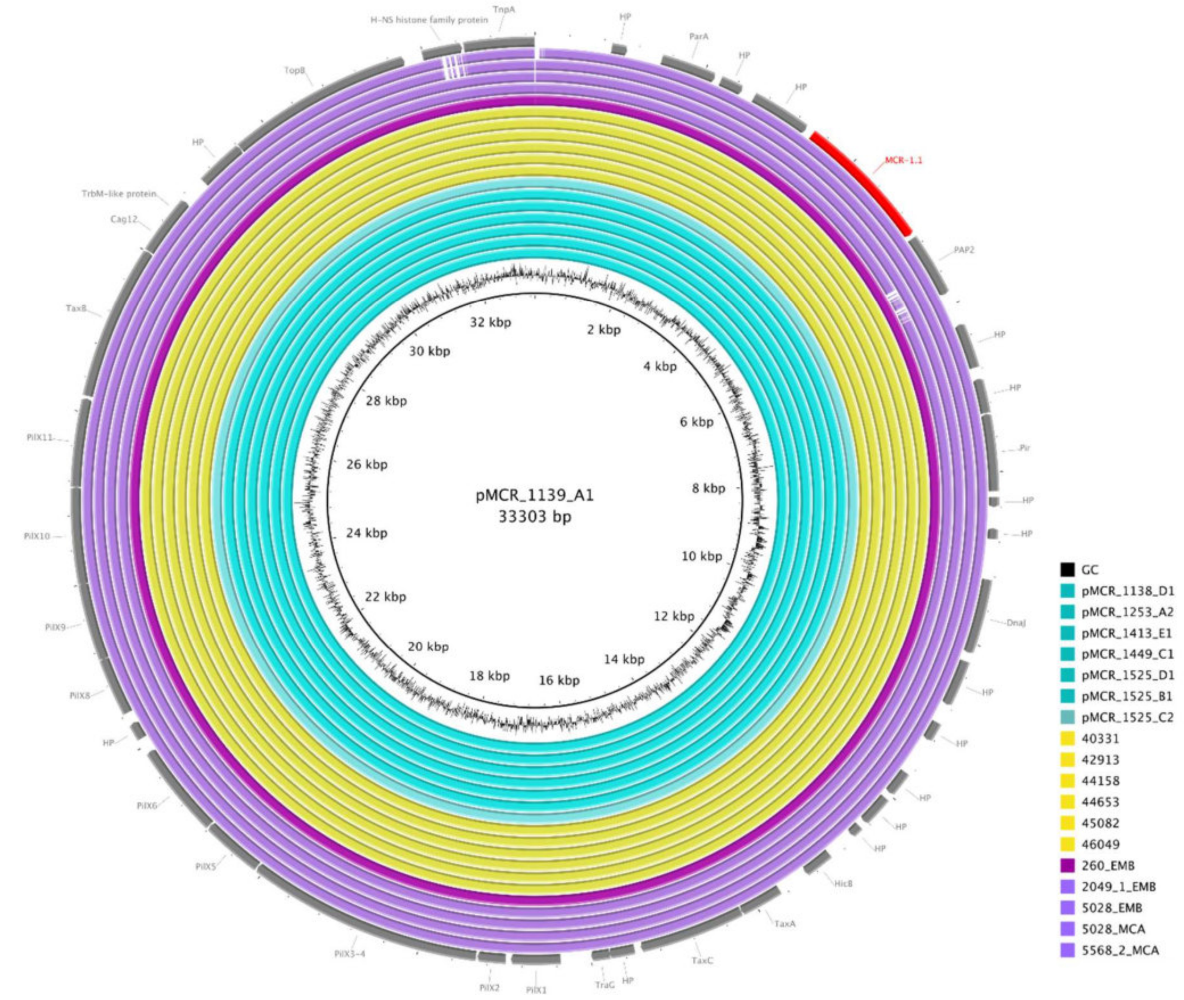

Figure S5. BRIG comparison of *mcr-1*-encoding IncX4 plasmids.

The plasmid pMCR_1139_A1 (GenBank n. NZ_MT929277.1) from *E. coli* ST10 isolated from raw turkey meat imported from Poland to the Czech Republic was used as a reference for the BRIG comparison. The light blue colour was used for the other plasmids originated from the same study: pMCR_1138_D1 (GenBank n. NZ_MT929276.1; *E. coli* ST744, raw turkey meat, Germany), pMCR_1253_A2 (GenBank n. NZ_MT929278.1; *E. coli* ST10, raw turkey meat, Germany), pMCR_1449_C1 (GenBank n. NZ_MT929279.1; *E. coli* ST1079, raw turkey liver, Brazil), pMCR_1413_E1 (GenBank n. NZ_MT929275.1; *E. coli* ST354, raw turkey meat, Czechia), pMCR_1525_B1 (GenBank n. NZ_MT929280.1; *K. pneumoniae* ST147, raw turkey liver, Brazil), pMCR_1525_C2 (GenBank n. NZ_MT929281.1; *K. pneumoniae* ST11, raw turkey liver, Brazil) and pMCR_1525_D1 (GenBank n. NZ_MT929282.1; *E. coli* ST349, raw turkey meat, Brazil). The yellow colour was used for six plasmids that originated in human isolates from the Czech Republic: pMCR-1-40331 (GenBank n. OP428973; *K. pneumoniae* ST290, urine), pMCR-1-42913 (GenBank n. OP428974; *E. coli* ST448, rectal swab), pMCR-1-44158 (GenBank n. OP428975; *E. coli* ST156, wound swab), pMCR-1-44563 (GenBank n.

OP428976; *E. coli* ST1196, urine), pMCR-1-45082 (GenBank n. OP428977; *E. coli* ST744, blood) and pMCR-1-46049 (GenBank n. OP428978; *K. pneumoniae* ST147, pus).
Eighty-one isolates of our study with the identical BRIG profile same as the reference plasmid pMCR_1139_A1 are represented by the isolate 260_EMB in dark purple colour. The other IncX4 from our study are shown in light purple colour: 2049/1_EMB (representing also 2049/1_MCA), 5028_EMB (representing also 817_MCA and 6031_EMB), 5028_MCA and 5568/2_MCA. HP, hypothetical protein

a)

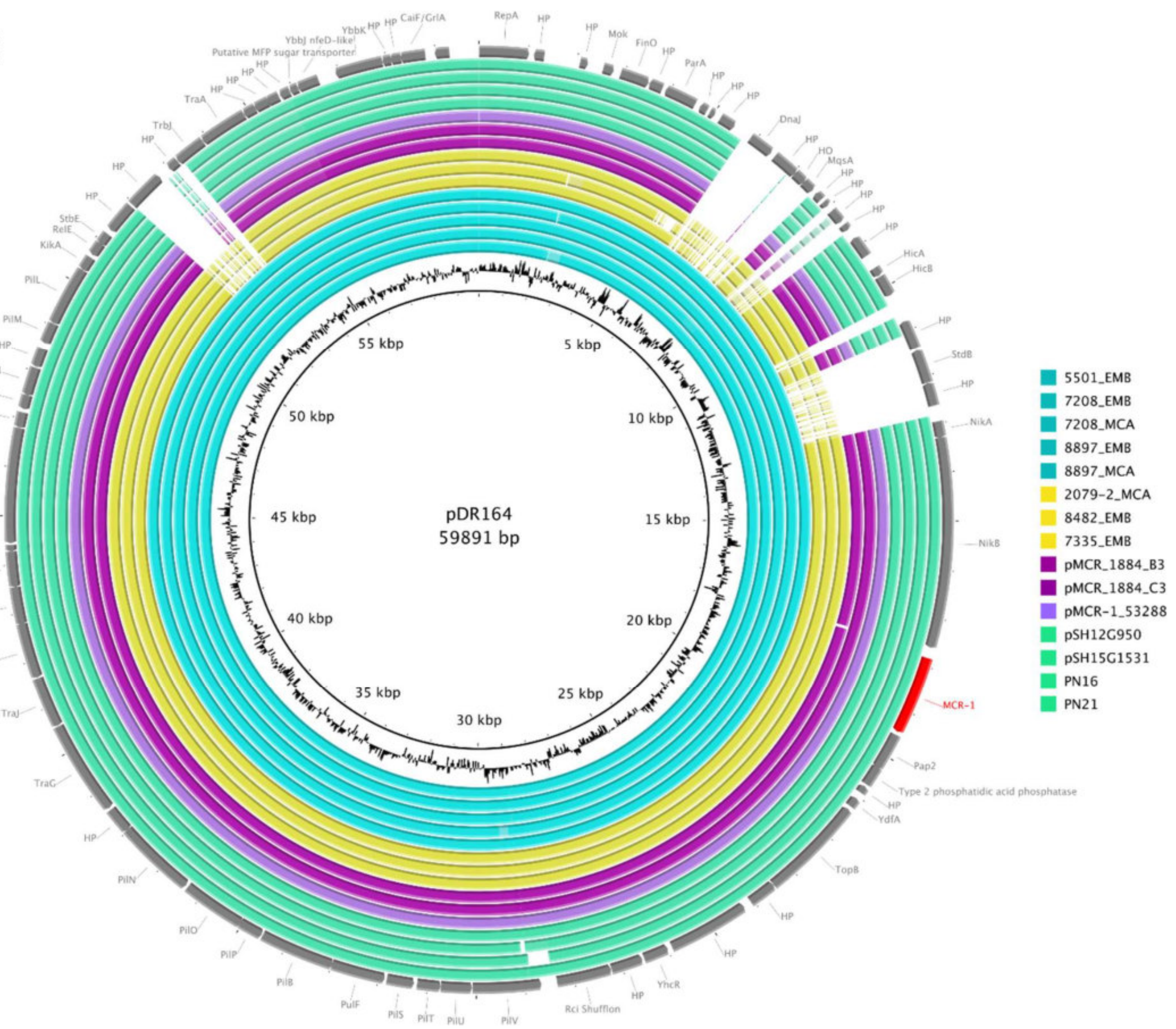

b)

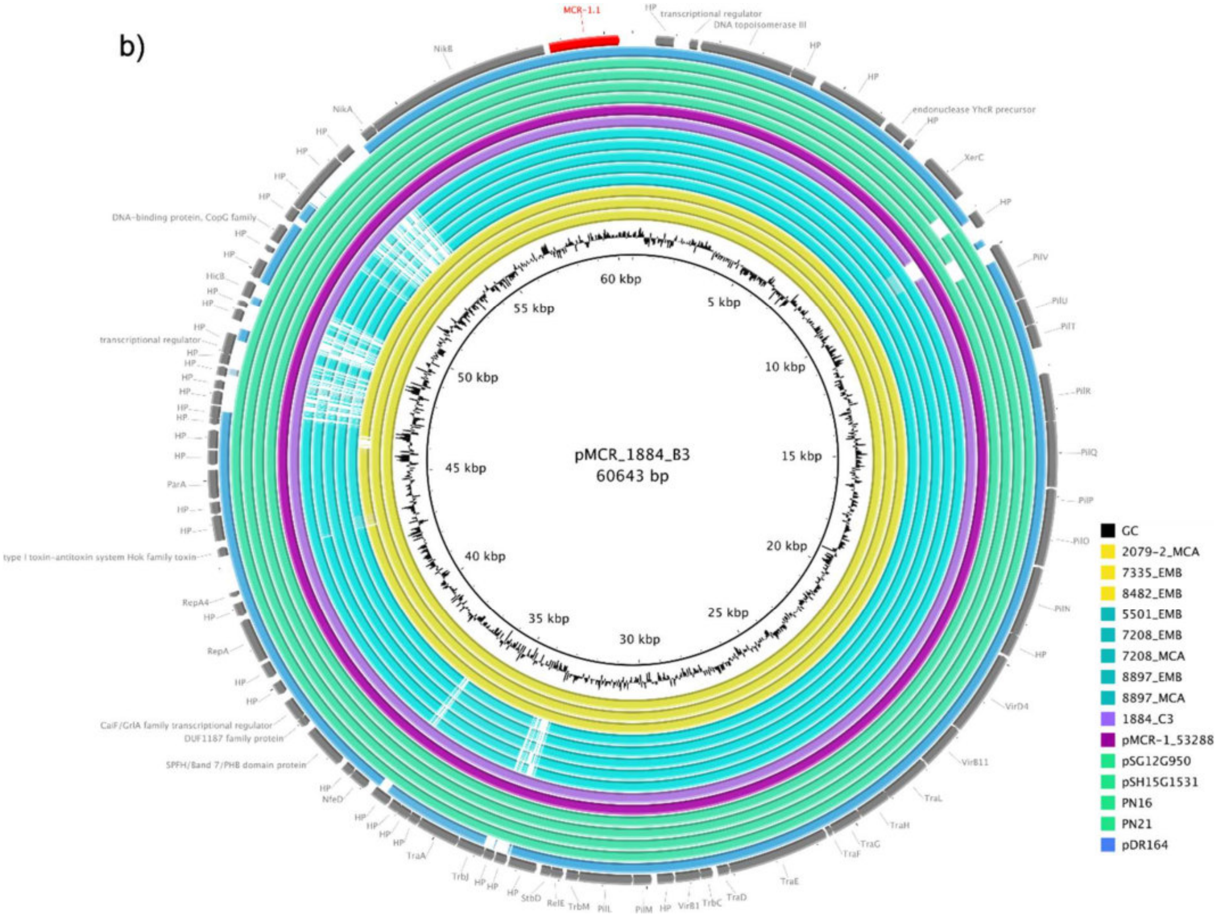

Figure S6. BRIG comparison of *mcr-1*-encoding IncI2 plasmids.

a) The plasmid pDR164 (GenBank n. MK542639.1) originated from *E. coli* ST2280 isolated from a black kite in Russia was used as a reference for the BRIG comparison.
b) The plasmid pMCR_1884_B3 (GenBank n. MT929283.1) originating from *E. coli* ST162 from imported rabbit meat (China) to the Czech Republic was used as a reference for the BRIG comparison.
The light blue colour (group I) and yellow colour (group II) was used for the other IncI2 plasmids originated from this study. The dark purple colour was used for plasmids originating from isolates obtained from imported meat to the Czech Republic: pMCR_1884_B3 (GenBank n. MT929283.1; *E. coli* ST162, rabbit meat, China) and pMCR_1884_C3 (GenBank n. MT929290.1; *C. braakii*, rabbit meat, China). Light purple colour was used for pMCR-1-53288 (GenBank n. OP434482) isolated from a clinical isolate of *E. coli* ST538. The green colour was used for similar IncI2 plasmids in GenBank database: pSH12G950 (GenBank n. MH522410.1; *Salmonella* Typhimurium, clinical, China), pSH15G1531 (GenBank n. MH522416.1; *Salmonella* Typhimurium, clinical, China), PN16 (GenBank n. MG489944.1; *E. coli*, chicken meat, Thailand) and PN21 (GenBank n. MG557851.1; *E. coli*, chicken feces, Thailand). Dark blue colour was used for plasmid pDR164 (GenBank n. MK542639.1, *E. coli* ST2280, black kite in Russia). HP, hypothetical protein

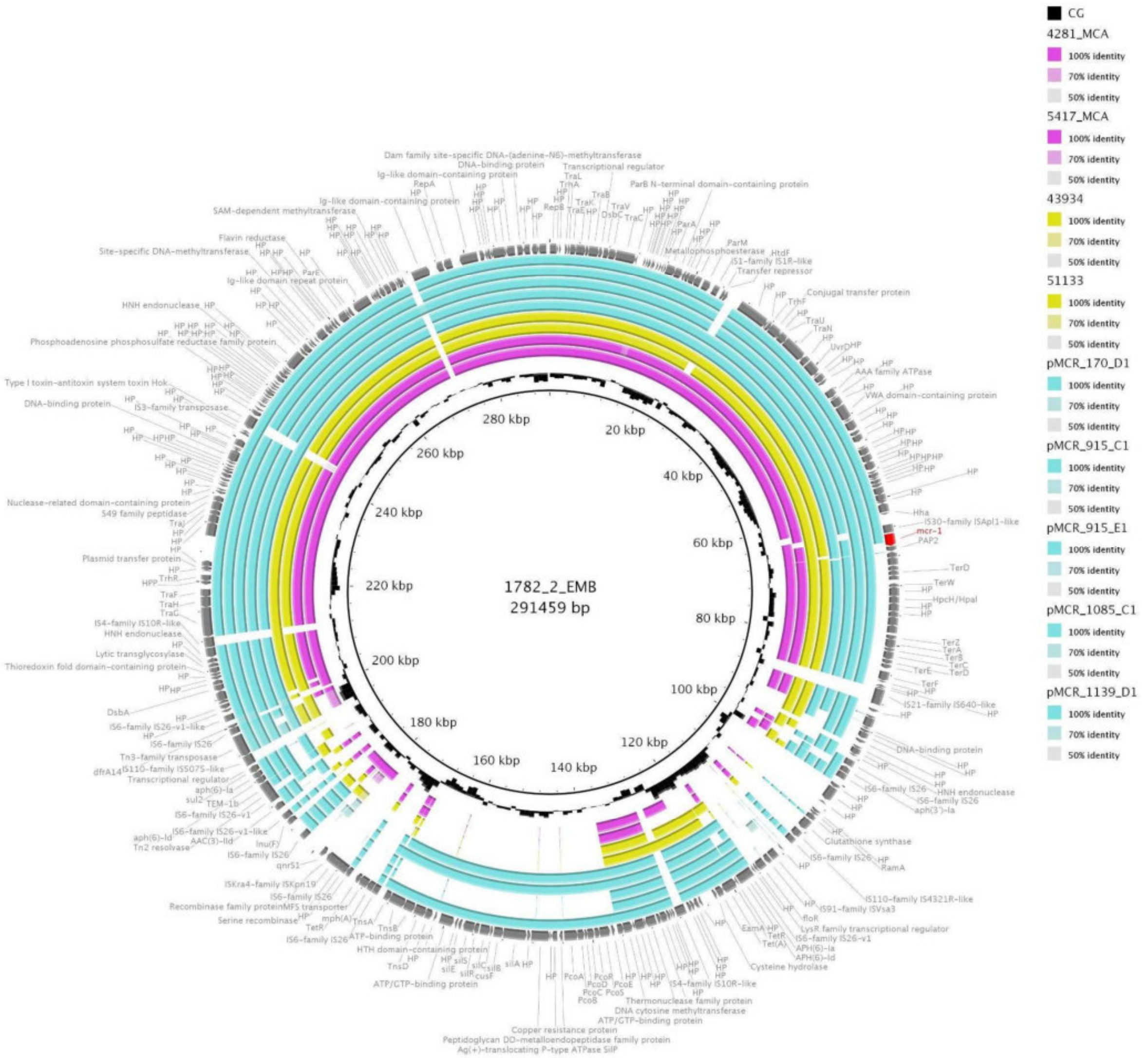

Figure S7. BRIG comparison of *mcr-1*-encoding IncHI2/ST4 plasmids originating from *E. coli* from the Czech Republic.

The complete plasmid p1782/2_EMB (GenBank n. OQ07808; 291,5 kb) of our study was used as a reference for the BRIG comparison. The other two complete plasmids from our study p4281_EMB (GenBank n. OQ078086; 230,6 kb) and p5417_MCA (GenBank n. OQ078087; 198 kb) are marked by purple colour. The yellow colour was used for the InHI2/ST4 plasmids isolated from clinical samples in the Czech Republic: pMCR1-43934 (GenBank n. OP950834; *E. coli* ST8186; 225,7 kb) and pMCR1-51133 (GenBank n. OP950835; *E. coli* ST117; 237,7 kb). The light blue colour was used for five HI2/ST4 plasmids that originated from raw turkey meat imported to the Czech Republic from Poland: pMCR_170_D1 (GenBank n. NZ_MT929288.1; *E. coli* ST7973; 284,2 kb), pMCR_915_C1 (GenBank n. NZ_MT929284.1; *E. coli* ST224; 253,1 kb), pMCR_915_E1 (GenBank n. NZ_MT929285.1; *E. coli* ST1140; 250,7 kb), pMCR_1085_C1 (GenBank n. NZ_MT929286.1; *E. coli* ST756; 251,5 kb) and pMCR_1139_D1 (GenBank n. NZ_MT929287.1; *E. coli* ST1167; 267,4 kb). HP, hypothetical protein.

Table S1: Characteristics of *E. coli* isolates with *mcr* genes

Isolate no	BioSample (GenBank accession)	Bacterial species	Selection media with col 3,5mg/L [a]	Species origin	Sample type	Sample origin [b]	Sampling place		Sampling year	MLST [c]	Phylogroup	mcr variant: *mcr-1.1*	*mcr-1.12*	*mcr-1.26*	Plasmids and conjugations [d]: Conjugative transfer	*mcr* location on plasmid
3180/1	SAMN31968641	*E.coli*	MCA	Gallus gallus	fresh meat	PL	ZS	Žďár nad Sázavou	2020	ST34	A	+			nt	IncX4
3180/1	SAMN31968642	*E.coli*	EMB	Gallus gallus	fresh meat	PL	ZS	Žďár nad Sázavou	2020	ST34	A	+			yes	IncX4
3286	SAMN31968643	*E.coli*	MCA	Gallus gallus	fresh meat	PL	M	Mělník	2020	ST2040	A	+			nt	IncX4
3286	SAMN31968644	*E.coli*	EMB	Gallus gallus	fresh meat	PL	M	Mělník	2020	ST7366	A	+			yes	IncX4
3423	SAMN31968645	*E.coli*	EMB	Gallus gallus	fresh meat	CZ	PRH	Praha	2020	ST48	A	+			yes	IncX4
3845	SAMN31968646	*E.coli*	MCA	Gallus gallus	fresh meat	CZ	SE	Semily	2020	ST57	E	+			nt	IncX4
3845	SAMN31968647	*E.coli*	EMB	Gallus gallus	fresh meat	CZ	SE	Semily	2020	ST997	E	+			yes	IncX4
4041	SAMN31968648	*E.coli*	MCA	Gallus gallus	fresh meat	CZ	US	Ústí nad Orlicí	2020	ST744	A	+			no	chromosome
4067	SAMN31968649	*E.coli*	MCA	Gallus gallus	fresh meat	PL	STR	Strakonice	2020	ST533	B1	+			nt	IncX4
4067	SAMN31968650	*E.coli*	EMB	Gallus gallus	fresh meat	PL	STR	Strakonice	2020	ST533	B1	+			nt	IncX4
4144	SAMN31968651	*E.coli*	MCA	Gallus gallus	fresh meat	CZ	UH	Uherské Hradiště	2020	ST410	C	+			yes	IncX4
4144	SAMN31968652	*E.coli*	EMB	Gallus gallus	fresh meat	CZ	UH	Uherské Hradiště	2020	ST191	A	+			nt	IncX4
4281	SAMN31968653	*E.coli*	EMB	Gallus gallus	fresh meat	CZ	PRE	Přerov	2020	ST641	B1	+			no	IncHI2
4282	SAMN31968654	*E.coli*	EMB	Gallus gallus	fresh meat	CZ	UH	Uherské Hradiště	2020	ST1011	E	+			nt	IncX4
4526	SAMN31968655	*E.coli*	MCA	Gallus gallus	fresh meat	PL	KR	Kroměříž	2020	ST641	B1	+			nt	IncX4
4526	SAMN31968656	*E.coli*	EMB	Gallus gallus	fresh meat	PL	KR	Kroměříž	2020	ST641	B1	+			yes	IncX4
4633	SAMN31968657	*E.coli*	MCA	Gallus gallus	fresh meat	CZ	TR	Třebíč	2020	ST1141	A	+			yes	IncX4
4633	SAMN31968658	*E.coli*	EMB	Gallus gallus	fresh meat	CZ	TR	Třebíč	2020	ST4204	A	+			nt	IncX4
4640	SAMN31968659	*E.coli*	MCA	Gallus gallus	fresh meat	PL	BRN	Brno-venkov	2020	ST1011	E	+			nt	IncX4
4640	SAMN31968660	*E.coli*	EMB	Gallus gallus	fresh meat	PL	BRN	Brno-venkov	2020	ST1011	E	+			nt	IncX4
4689	SAMN31968661	*E.coli*	EMB	Gallus gallus	fresh meat	CZ	PAR	Pardubice	2020	ST624	F	+			yes	IncX4
4804	SAMN31968662	*E.coli*	EMB	Gallus gallus	fresh meat	CZ	BE	Benešov	2020	ST1011	E	+			nt	IncX4
5027	SAMN31968663	*E.coli*	MCA	Gallus gallus	fresh meat	CZ	FM	Frýdek-Místek	2020	ST57	E	+			yes	IncX4
5027	SAMN31968664	*E.coli*	EMB	Gallus gallus	fresh meat	CZ	FM	Frýdek-Místek	2020	ST624	F	+			nt	IncX4
5028	SAMN31968665	*E.coli*	MCA	Gallus gallus	fresh meat	PL	OS	Ostrava-město	2020	ST10	A	+			nt	IncX4
5028	SAMN31968666	*E.coli*	EMB	Gallus gallus	fresh meat	PL	OS	Ostrava-město	2020	ST10	A	+			yes	IncX4
5391	SAMN31968667	*E.coli*	MCA	Gallus gallus	fresh meat	PL	KR	Kroměříž	2020	ST155	B1	+			yes	IncX4
5391	SAMN31968668	*E.coli*	EMB	Gallus gallus	fresh meat	PL	KR	Kroměříž	2020	ST1011	E	+			nt	IncX4
5417	SAMN31968669	*E.coli*	MCA	Gallus gallus	fresh meat	CZ	STR	Strakonice	2020	ST162	B1	+			yes	IncHI2
5417	SAMN31968670	*E.coli*	EMB	Gallus gallus	fresh meat	CZ	STR	Strakonice	2020	ST162	B1	+			yes	IncHI2
5501	SAMN31968671	*E.coli*	MCA	Gallus gallus	fresh meat	CZ	VS	Vsetín	2020	ST93	A	+			nt	IncX4
5501	SAMN31968672	*E.coli*	EMB	Gallus gallus	fresh meat	CZ	VS	Vsetín	2020	ST744	A	+			yes	IncI2
5533	SAMN31968673	*E.coli*	MCA	Gallus gallus	fresh meat	CZ	KA	Karviná	2020	ST162	B1	+			yes	IncX4
5533	SAMN31968674	*E.coli*	EMB	Gallus gallus	fresh meat	CZ	KA	Karviná	2020	ST533	B1	+			yes	IncX4
5568/2	SAMN31968675	*E.coli*	MCA	Gallus gallus	fresh meat	CZ	BER	Beroun	2020	ST6728	A	+			yes	IncX4
5568/2	SAMN31968676	*E.coli*	EMB	Gallus gallus	fresh meat	CZ	BER	Beroun	2020	ST58	B1	+			yes	IncX4
5674	SAMN31968677	*E.coli*	MCA	Gallus gallus	fresh meat	PL	JN	Jablonec nad Nisou	2020	ST1011	E	+			yes	IncX4
5674	SAMN31968678	*E.coli*	EMB	Gallus gallus	fresh meat	PL	JN	Jablonec nad Nisou	2020	ST1011	E	+			nt	IncX4
6029	SAMN31968679	*E.coli*	MCA	Gallus gallus	fresh meat	CZ	PRO	Prostějov	2020	ST10	A	+			no	IncX4
6029	SAMN31968680	*E.coli*	EMB	Gallus gallus	fresh meat	CZ	PRO	Prostějov	2020	ST10	A	+			nt	IncX4
6031	SAMN31968681	*E.coli*	EMB	Gallus gallus	fresh meat	UKR	MB	Mladá Boleslav	2020	ST155	B1	+			nt	IncX4
6406	SAMN31968682	*E.coli*	EMB	Gallus gallus	fresh meat	CZ	NJ	Nový Jičín	2020	ST48	A	+			yes	IncX4
6645	SAMN31968685	*E.coli*	MCA	Gallus gallus	fresh meat	PL	JI	Jičín	2020	ST131	B2	+			yes	IncX4
6645	SAMN31968686	*E.coli*	EMB	Gallus gallus	fresh meat	PL	JI	Jičín	2020	ST131	B2	+			nt	IncX4
6646	SAMN31968687	*E.coli*	MCA	Gallus gallus	fresh meat	CZ	NA	Náchod	2020	ST48	A	+			no	chromosome
6646	SAMN31968688	*E.coli*	EMB	Gallus gallus	fresh meat	CZ	NA	Náchod	2020	ST48	A	+			no	chromosome
7065	SAMN31968689	*E.coli*	MCA	Gallus gallus	fresh meat	CZ	KR	Kroměříž	2020	ST162	B1	+			no	chromosome
7065	SAMN31968690	*E.coli*	EMB	Gallus gallus	fresh meat	CZ	KR	Kroměříž	2020	ST2705	A	+			nt	IncX4
7168	SAMN31968691	*E.coli*	MCA	Gallus gallus	fresh meat	CZ	RK	Rychnov nad Kněžnou	2020	ST40	B1	+			nt	IncX4
7168	SAMN31968692	*E.coli*	EMB	Gallus gallus	fresh meat	CZ	RK	Rychnov nad Kněžnou	2020	ST361	A	+			no	IncX4
7208	SAMN31968693	*E.coli*	MCA	Gallus gallus	fresh meat	PL	OS	Ostrava-město	2020	ST162	B1	+			yes	IncI2
7208	SAMN31968694	*E.coli*	EMB	Gallus gallus	fresh meat	PL	OS	Ostrava-město	2020	ST162	B1	+			no	IncI2
7335	SAMN31968695	*E.coli*	MCA	Gallus gallus	fresh meat	CZ	SE	Semily	2020	ST453	B1	+			yes	IncX4
7335	SAMN31968696	*E.coli*	EMB	Gallus gallus	fresh meat	CZ	SE	Semily	2020	ST58	B1	+			yes	IncI2
7410	SAMN31968697	*E.coli*	MCA	Gallus gallus	fresh meat	CZ	SUM	Šumperk	2020	ST1011	E	+			nt	IncX4
7410	SAMN31968698	*E.coli*	EMB	Gallus gallus	fresh meat	CZ	SUM	Šumperk	2020	ST1011	E	+			nt	IncX4
7506	SAMN31968699	*E.coli*	MCA	Gallus gallus	fresh meat	CZ	FM	Frýdek-Místek	2020	ST1011	E	+			yes	IncHI2
7506	SAMN31968700	*E.coli*	EMB	Gallus gallus	fresh meat	CZ	FM	Frýdek-Místek	2020	ST162	B1	+			nt	IncX4
7674	SAMN31968701	*E.coli*	MCA	Gallus gallus	fresh meat	PL	KA	Karviná	2020	ST162	B1	+			nt	IncX4
7674	SAMN31968702	*E.coli*	EMB	Gallus gallus	fresh meat	PL	KA	Karviná	2020	ST744	A	+			yes	IncX4
7727	SAMN31968703	*E.coli*	MCA	Gallus gallus	fresh meat	CZ	SUM	Šumperk	2020	ST744	A	+			yes	IncX4
7727	SAMN31968704	*E.coli*	EMB	Gallus gallus	fresh meat	CZ	SUM	Šumperk	2020	ST744	A	+			yes	IncX4
7837	SAMN31968705	*E.coli*	MCA	Gallus gallus	fresh meat	PL	OP	Opava	2020	ST93	A	+			yes	IncHI2
7837	SAMN31968706	*E.coli*	EMB	Gallus gallus	fresh meat	PL	OP	Opava	2020	ST744	A	+			nt	IncX4
8211	SAMN31968707	*E.coli*	MCA	Gallus gallus	fresh meat	CZ	HB	Havlíčkův Brod	2020	ST154	B1	+			nt	IncX4
8211	SAMN31968708	*E.coli*	EMB	Gallus gallus	fresh meat	CZ	HB	Havlíčkův Brod	2020	ST154	A	+			yes	IncX4
8482	SAMN31968709	*E.coli*	MCA	Gallus gallus	fresh meat	CZ	BRN	Brno-venkov	2020	ST10	A	+			nt	IncX4
8482	SAMN31968710	*E.coli*	EMB	Gallus gallus	fresh meat	CZ	BRN	Brno-venkov	2020	ST58	B1	+			nt	IncI2
8563	SAMN31968711	*E.coli*	MCA	Gallus gallus	fresh meat	PL	NA	Náchod	2020	ST7941	A	+			yes	IncX4
8563	SAMN31968712	*E.coli*	EMB	Gallus gallus	fresh meat	PL	NA	Náchod	2020	ST7941	A	+			yes	IncX4
8564	SAMN31968713	*E.coli*	MCA	Gallus gallus	fresh meat	CZ	JI	Jičín	2020	ST162	B1	+			nt	IncX4
8564	SAMN31968714	*E.coli*	EMB	Gallus gallus	fresh meat	CZ	JI	Jičín	2020	ST162	B1	+			nt	IncX4
8615	SAMN31968715	*E.coli*	MCA	Gallus gallus	fresh meat	CZ	TR	Třebíč	2020	ST93	A	+			nt	IncX4
8615	SAMN31968716	*E.coli*	EMB	Gallus gallus	fresh meat	CZ	TR	Třebíč	2020	ST93	A	+			nt	IncX4
8622	SAMN31968717	*E.coli*	MCA	Gallus gallus	fresh meat	CZ	SUM	Šumperk	2020	ST93	A	+			yes	IncX4
8693	SAMN31968718	*E.coli*	EMB	Gallus gallus	fresh meat	CZ	VS	Vsetín	2020	ST10	A	+			nt	IncX4
8799	SAMN31968719	*E.coli*	MCA	Gallus gallus	fresh meat	CZ	BRU	Bruntál	2021	ST1011	E	+			nt	IncX4
8799	SAMN31968720	*E.coli*	EMB	Gallus gallus	fresh meat	CZ	BRU	Bruntál	2021	ST1011	E	+			nt	IncX4
8815	SAMN31968721	*E.coli*	MCA	Gallus gallus	fresh meat	PL	US	Ústí nad Orlicí	2021	ST118	E	+			yes	IncX4
8815	SAMN31968722	*E.coli*	EMB	Gallus gallus	fresh meat	PL	US	Ústí nad Orlicí	2021	ST117	G	+			yes	IncX4
8897	SAMN31968723	*E.coli*	MCA	Gallus gallus	fresh meat	PL	JES	Jeseník	2021	ST162	B1	+			nt	IncI2
8897	SAMN31968724	*E.coli*	EMB	Gallus gallus	fresh meat	PL	JES	Jeseník	2021	ST48	A	+			yes	IncI2
8950	SAMN31968725	*E.coli*	MCA	Gallus gallus	fresh meat	CZ	KO	Kolín	2021	ST1011	E	+			nt	IncX4
8950	SAMN31968726	*E.coli*	EMB	Gallus gallus	fresh meat	CZ	KO	Kolín	2021	ST1011	E	+			nt	IncX4
8996	SAMN31968727	*E.coli*	MCA	Gallus gallus	fresh meat	CZ	NJ	Nový Jičín	2021	ST359	B1	+			nt	IncX4
8996	SAMN31968728	*E.coli*	EMB	Gallus gallus	fresh meat	CZ	NJ	Nový Jičín	2021	ST359	B1	+			nt	IncX4
3959	SAMN31968729	*E.coli*	EMB	Gallus gallus	caecum	CZ	STR	Strakonice	2020	ST1196	B1	+			nt	IncX4
4063	SAMN31968730	*E.coli*	MCA	Gallus gallus	caecum	CZ	BE	Benešov	2020	ST10	A	+			yes	IncHI2
4063	SAMN31968731	*E.coli*	EMB	Gallus gallus	caecum	CZ	BE	Benešov	2020	ST10	A	+			yes	IncHI2
7066	SAMN31968732	*E.coli*	MCA	Gallus gallus	caecum	CZ	SVI	Svitavy	2020	ST2705	A	+			yes	IncX4
260	SAMN31968733	*E.coli*	MCA	Bos taurus	fresh meat	CZ	HB	Havlíčkův Brod	2021	ST746	A	+			nt	IncX4
260	SAMN31968734	*E.coli*	EMB	Bos taurus	fresh meat	CZ	HB	Havlíčkův Brod	2021	ST361	A	+			yes	IncX4
279	SAMN31968735	*E.coli*	MCA	Bos taurus	fresh meat	DE	VS	Vsetín	2021	ST345	B1	+			yes	IncX4
279	SAMN31968736	*E.coli*	EMB	Bos taurus	fresh meat	DE	VS	Vsetín	2021	ST69	D		+		yes	IncX4
699	SAMN31968737	*E.coli*	EMB	Bos taurus	fresh meat	CZ	NA	Náchod	2021	ST10	A	+			nt	IncX4
2008/1	SAMN31968738	*E.coli*	MCA	Bos taurus	fresh meat	CZ	STR	Strakonice	2021	ST428	B2	+			nt	IncX4
2049/2	SAMN31968739	*E.coli*	MCA	Bos taurus	fresh meat	CZ	VS	Vsetín	2021	ST58	B1	+			nt	IncX4
734	SAMN31968740	*E.coli*	MCA	Sus scrofa	fresh meat	CZ	NA	Náchod	2021	ST88	C	+			nt	IncX4
734	SAMN31968741	*E.coli*	EMB	Sus scrofa	fresh meat	CZ	NA	Náchod	2021	ST88	C	+			nt	IncX4
817	SAMN31968742	*E.coli*	MCA	Sus scrofa	fresh meat	CZ	PAR	Pardubice	2021	ST93	A	+			nt	IncX4
817	SAMN31968743	*E.coli*	EMB	Sus scrofa	fresh meat	CZ	PAR	Pardubice	2021	ST1485	F	+			no	chromosome
1357	SAMN31968744	*E.coli*	MCA	Sus scrofa	fresh meat	CZ	JH	Jindřichův Hradec	2021	ST1011	E	+			nt	IncX4
1782/2	SAMN31968745	*E.coli*	EMB	Sus scrofa	fresh meat	CZ	TE	Teplice	2021	ST93	A	+			yes	IncHI2
2049/1	SAMN31968746	*E.coli*	MCA	Sus scrofa	fresh meat	CZ	VS	Vsetín	2021	ST162	B1			+	yes	IncX4
2049/1	SAMN31968747	*E.coli*	EMB	Sus scrofa	fresh meat	CZ	VS	Vsetín	2021	ST162	B1			+	yes	IncX4
2079/2	SAMN31968748	*E.coli*	MCA	Sus scrofa	fresh meat	CZ	OL	Olomouc	2021	ST155	B1	+			nt	IncI2
BA 397/4	SAMN31968749	*E.coli*	MCA	Sus scrofa	caecum	CZ	KO	Kolín	2021	ST1011	E	+			nt	IncX4
BA 1137/4	SAMN31968750	*E.coli*	MCA	Sus scrofa	caecum	CZ	NA	Náchod	2021	ST1721	A	+			nt	IncX4
BA 1137/4	SAMN31968751	*E.coli*	EMB	Sus scrofa	caecum	CZ	NA	Náchod	2021	ST58*	B1	+			nt	IncX4

Antibiotic resistance profile [e]

BioSample	COL MIC >2 mg/ml	AMP	S	S3	TE	SXT	C	KZ	NA	CAZ	CN	AMC	CIP	FOS	ETP	IMP	MEM	ATM	F	AZM	TGC
SAMN31968641	8	6	12	6	6	6	20	19	6	25	15	15	32	28	28	25	27	30	17	6	19
SAMN31968642	8	6	10	6	6	6	6	16	6	25	14	20	6	25	30	25	30	28	14	17	17
SAMN31968643	4	6	11	18	20	23	23	18	6	18	6	15	17	28	26	25	25	25	17	25	20
SAMN31968644	4	6	10	6	6	6	6	16	6	25	15	13	10	26	29	25	26	27	16	21	16
SAMN31968645	8	6	10	6	6	6	22	17	6	25	15	17	25	26	28	25	28	26	17	12	18
SAMN31968646	8	6	6	6	6	6	23	15	20	23	11	10	27	25	26	25	24	26	16	13	19
SAMN31968647	8	6	6	6	6	6	8	16	6	24	13	6	10	24	29	28	28	26	17	17	19
SAMN31968648	8	6	11	15	6	23	22	14	6	20	16	11	9	26	26	24	25	26	16	23	18
SAMN31968649	4	6	8	6	6	6	19	18	6	29	17	16	6	25	32	27	27	29	17	15	20
SAMN31968650	4	6	11	6	6	6	21	16	6	27	17	16	9	28	31	22	27	28	16	21	21
SAMN31968651	8	6	6	6	6	6	20	20	6	28	16	18	6	29	30	27	28	31	19	15	23
SAMN31968652	8	6	12	6	6	6	22	15	6	24	15	14	20	30	30	23	24	26	19	21	19
SAMN31968653	8	6	6	6	6	6	6	11	6	22	15	14	20	30	28	24	28	25	17	18	18
SAMN31968654	8	6	6	6	6	22	6	6	6	21	6	6	11	25	29	20	25	19	18	16	21
SAMN31968655	8	6	12	16	6	24	6	10	6	22	20	10	10	30	27	27	26	25	17	10	17
SAMN31968656	8	6	12	14	6	24	6	14	6	26	18	15	16	32	27	22	24	23	18	10	17
SAMN31968657	4	6	12	21	17	25	17	19	6	23	14	19	25	26	26	25	24	27	16	13	20
SAMN31968658	8	6	6	6	18	6	22	6	6	24	15	21	6	29	30	27	27	28	15	12	20
SAMN31968659	4	6	6	6	6	6	22	14	6	22	6	6	9	25	23	26	25	27	17	14	19
SAMN31968660	8	6	6	6	6	6	21	14	6	24	6	16	10	25	30	25	29	28	18	24	19
SAMN31968661	8	6	6	6	6	6	22	15	6	24	16	17	10	30	29	26	29	28	15	22	21
SAMN31968662	8	6	6	6	6	6	20	14	6	20	14	18	9	24	28	25	22	28	18	22	17
SAMN31968663	8	6	6	20	20	26	23	15	6	23	20	14	12	26	28	25	26	25	15	25	20
SAMN31968664	8	6	6	18	23	6	23	17	6	23	20	20	10	26	32	30	30	28	15	23	23
SAMN31968665	8	6	6	19	6	24	22	14	6	22	15	17	14	26	24	24	25	27	15	25	20
SAMN31968666	8	6	11	21	6	26	27	13	24	27	16	18	35	28	32	28	32	30	16	26	22
SAMN31968667	4	6	6	19	6	24	21	17	6	22	18	6	16	28	26	24	25	25	16	22	19
SAMN31968668	8	6	6	6	6	23	6	6	6	22	6	8	11	30	30	26	30	14	18	16	22
SAMN31968669	4	6	6	6	6	6	23	15	6	29	21	10	11	35	35	27	30	32	17	25	23
SAMN31968670	4	6	6	6	6	6	25	15	6	26	10	6	10	33	30	25	30	30	18	24	22
SAMN31968671	8	6	11	6	6	6	22	11	6	22	16	17	9	28	27	25	24	26	16	21	19
SAMN31968672	8	6	6	6	6	6	24	15	6	27	15	15	13	30	30	28	30	30	18	6	25
SAMN31968673	4	6	6	6	6	6	23	16	6	24	15	19	11	29	26	25	27	28	16	23	21
SAMN31968674	8	6	6	6	6	6	20	15	6	25	15	14	6	28	30	25	28	26	16	23	22
SAMN31968675	4	6	12	6	6	6	22	15	6	23	17	16	6	27	25	22	25	26	16	23	18
SAMN31968676	4	6	12	17	6	25	20	17	6	25	6	16	18	28	33	28	30	30	15	13	20
SAMN31968677	8	6	6	6	6	6	9	11	6	22	16	6	10	23	25	24	23	26	17	22	19
SAMN31968678	8	6	6	6	6	6	6	15	6	28	20	6	11	27	32	28	32	30	18	20	21
SAMN31968679	8	6	6	6	6	6	19	15	6	22	14	17	6	26	27	25	25	28	13	23	20
SAMN31968680	8	6	6	6	6	6	23	17	6	28	17	15	6	27	32	30	30	30	16	25	23
SAMN31968681	16	6	6	6	6	6	6	6	6	27	6	17	12	6	30	18	27	30	15	10	21
SAMN31968682	8	6	13	15	6	8	25	17	6	28	19	17	12	29	32	28	31	34	17	25	22
SAMN31968685	8	6	11	21	20	25	22	13	20	22	16	16	25	26	26	22	21	25	17	20	17
SAMN31968686	8	6	12	23	21	28	18	16	23	28	18	14	32	23	32	27	30	31	20	20	22
SAMN31968687	8	6	7	6	6	6	22	16	6	25	15	16	6	23	29	25	25	26	15	13	21
SAMN31968688	8	6	8	6	8	6	25	17	6	28	15	16	9	25	32	27	32	30	16	17	23
SAMN31968689	8	6	11	17	6	24	22	6	6	15	16	18	16	37	23	25	25	8	17	25	21
SAMN31968690	8	6	13	23	6	28	13	20	18	23	17	16	18	32	33	27	31	30	13	21	20
SAMN31968691	4	6	11	6	6	6	8	15	17	24	15	15	21	30	28	27	25	28	17	12	21
SAMN31968692	4	6	13	16	21	28	23	21	6	28	13	19	12	33	31	27	30	30	18	15	22
SAMN31968693	4	6	6	6	6	6	6	16	6	24	15	18	11	30	25	24	26	7	21	6	20
SAMN31968694	8	6	6	6	6	6	6	6	6	9	16	21	14	33	27	27	29	6	23	8	22
SAMN31968695	4	6	9	6	6	6	6	12	6	24	15	15	10	27	29	25	24	25	16	16	18
SAMN31968696	8	6	12	6	6	6	6	15	6	25	16	17	13	28	30	26	28	28	15	14	23
SAMN31968697	8	6	6	6	7	21	6	6	6	12	6	8	9	22	23	21	26	13	17	16	19
SAMN31968698	4	6	6	6	8	24	6	6	6	12	6	8	10	24	26	25	29	15	18	18	20
SAMN31968699	8	6	6	6	6	6	21	13	6	24	6	17	10	24	29	24	26	28	18	23	19
SAMN31968700	4	6	11	6	6	6	6	15	6	20	16	16	11	28	31	26	28	28	17	18	19
SAMN31968701	8	6	6	6	6	6	6	16	6	24	14	18	11	31	26	25	26	6	21	6	21
SAMN31968702	8	6	6	6	6	6	6	17	6	24	17	18	10	31	31	26	27	27	17	11	16
SAMN31968703	16	6	11	6	6	23	20	18	6	22	16	6	6	28	28	26	27	25	18	14	19
SAMN31968704	8	6	13	6	6	23	21	19	6	22	16	6	8	29	29	26	29	29	18	18	19
SAMN31968705	4	6	6	6	6	6	6	15	16	25	20	6	19	27	28	23	27	28	18	28	20
SAMN31968706	8	6	6	6	6	6	6	16	6	24	6	6	10	28	29	25	29	27	21	26	20
SAMN31968707	8	6	6	6	6	6	23	15	18	26	15	14	28	28	28	25	26	30	17	23	22
SAMN31968708	8	6	6	6	6	6	6	19	6	24	15	15	8	30	31	26	30	28	20	20	19
SAMN31968709	8	6	6	6	6	6	20	17	8	24	15	13	29	27	30	25	26	28	16	12	18
SAMN31968710	4	6	10	6	6	6	8	17	6	25	16	18	12	28	31	25	27	26	15	12	20
SAMN31968711	8	6	6	6	6	6	6	15	6	24	6	15	6	26	28	24	27	27	17	12	18
SAMN31968712	8	6	6	6	6	6	6	14	6	25	6	15	6	29	30	27	28	28	19	12	19
SAMN31968713	8	6	6	6	19	6	6	19	6	25	6	6	12	30	30	27	30	30	17	13	21
SAMN31968714	8	6	6	6	19	6	6	15	6	28	16	6	10	31	30	27	31	30	19	24	20
SAMN31968715	8	6	6	6	6	6	25	17	6	26	16	19	13	24	30	26	30	30	15	20	20
SAMN31968716	8	6	6	6	6	6	20	19	6	21	16	19	12	27	31	26	31	27	16	16	20
SAMN31968717	8	6	6	6	6	6	6	6	6	18	16	18	12	26	27	25	28	28	15	20	21
SAMN31968718	16	6	11	6	20	6	22	18	6	24	15	20	20	28	29	26	29	29	20	13	21
SAMN31968719	8	6	6	6	6	6	20	20	6	23	15	17	9	24	30	25	27	27	17	21	19
SAMN31968720	16	6	6	6	6	6	21	16	6	25	16	18	10	22	27	22	26	28	18	21	19
SAMN31968721	16	6	12	20	6	24	21	15	6	24	19	16	20	21	29	29	28	28	18	22	19
SAMN31968722	16	6	6	6	6	6	6	22	6	30	8	9	6	37	33	28	25	30	19	12	18
SAMN31968723	8	6	10	6	6	27	6	6	6	13	16	20	13	32	27	27	27	6	20	22	19
SAMN31968724	8	6	6	6	21	21	25	6	6	21	15	19	8	29	28	25	29	15	12	6	21
SAMN31968725	8	6	11	6	6	6	6	15	6	27	20	17	11	25	32	29	32	32	19	26	22
SAMN31968726	8	6	10	6	6	6	6	16	6	26	15	19	10	25	22	24	27	23	18	12	20
SAMN31968727	8	6	6	6	6	20	6	10	6	23	20	14	10	27	29	26	30	27	14	24	20
SAMN31968728	8	6	6	6	6	17	6	16	6	22	14	15	10	28	40	26	29	28	16	13	20
SAMN31968729	4	6	13	6	6	6	6	19	6	26	16	6	6	30	29	27	31	28	19	19	20
SAMN31968730	8	6	6	6	6	6	22	13	6	22	19	6	23	25	20	20	6	6	15	22	19
SAMN31968731	8	6	6	6	6	6	23	16	6	25	16	16	23	26	30	25	30	27	18	19	19
SAMN31968732	16	6	12	20	6	25	21	16	20	24	16	16	24	31	27	26	25	23	13	11	12
SAMN31968733	8	6	6	21	6	27	24	6	22	25	16	18	38	28	30	25	30	27	16	11	20
SAMN31968734	4	6	11	6	12	6	20	17	6	20	16	16	11	27	28	23	28	27	19	13	20
SAMN31968735	16	6	6	6	6	6	6	15	6	20	15	16	8	30	30	27	28	27	17	23	20
SAMN31968736	8	6	6	6	6	6	6	17	6	25	14	19	10	26	28	24	28	29	19	12	19
SAMN31968737	8	6	6	6	6	20	26	17	21	29	16	17	24	27	32	30	33	30	18	19	22
SAMN31968738	8	6	6	6	6	20	6	12	11	22	15	15	16	30	30	29	30	32	21	15	20
SAMN31968739	8	6	6	6	6	6	6*	17	6	26	16	17	11	28	29	25	27	30	19	13	23
SAMN31968740	8	6	6	6	6	6	6	12	6	25	20	15	12	25	27	24	26	27	19	6	12
SAMN31968741	8	6	6	6	6	6	6	11	6	25	12	15	20	24	30	24	24	26	19	23	22
SAMN31968742	8	6	12	23	6	6	23	18	24	28	18	19	30	30	28	27	27	29	19	12	22
SAMN31968743	16	6	6	6	22	16	18	16	18	23	15	17	19	29	29	27	28	27	17	14	22
SAMN31968744	8	6	6	6	24	6	7	20	6	22	16	19	10	24	28	24	28	27	19	16	20
SAMN31968745	16	6	6	6	6	6	6	12	6	22	6	17	6	24	30	27	29	29	15	8	19
SAMN31968746	8	6	6	6	6	6	28	15	6	26	15	15	11	29	30	25	26	29	19	12	15
SAMN31968747	8	6	6	6	6	6	23	17	6	22	16	16	12	31	29	25	27	27	16	14	22
SAMN31968748	8	6	11	6	6	6	7	17	14	24	17	17	17	28	30	26	29	29	19	15	17
SAMN31968749	8	6	9	6	6	6	6	16	6	23	15	6	12	23	28	23	28	31	20	14	21
SAMN31968750	8	6	6	6*	6	6*	6*	18	6*	26	16	16	10*	25	29	26	28	28	17	15	16
SAMN31968751	4	6	6	6	22	6	21	17	22	21	16	15	27	25	30	25	27	25	16	14	24

BioSample	pMLST [g]	colV+	cvaA	cvaB	cvaC	cvi
SAMN31968641						
SAMN31968642						
SAMN31968643	[F18:A-:B1]	+	+			
SAMN31968644	[F18:A-:B8]	+				
SAMN31968645	[F-:A-:B20]					
SAMN31968646	[F24:A-:B1], IncI1/ST36 CC3	+				+
SAMN31968647	[F18:A-:B1], IncI1/ST29 CC2	+	+			
SAMN31968648	[F18:A6:B1], IncI1/ST36 CC3	+				+
SAMN31968649	[F1:A1:B1]					
SAMN31968650	[F1:A1:B1]					
SAMN31968651	[F18:A-:B1]	+	+	+	+	+
SAMN31968652	[F-:A:B-], IncI1/ST3* CC3					
SAMN31968653	[F18:A-:B1], IncHI2/ST4	+				+
SAMN31968654	[F24:A-:B1]	+	+	+	+	+
SAMN31968655	[F72:A-:B77], IncI1/ST3 CC3					
SAMN31968656	[F72:A-:B77], IncI1/ST3 CC3					
SAMN31968657						
SAMN31968658	[F18:A6:B8]	+				
SAMN31968659	[F24:A-:B1]					
SAMN31968660	[F24:A-:B1]					
SAMN31968661	[F2:A-:B1]	+	+	+	+	+
SAMN31968662	[F24:A-:B1]	+			+	+
SAMN31968663	[F18:A-:B1]	+	+	+		+
SAMN31968664	[F2:A-:B1]	+	+	+	+	+
SAMN31968665	[F2:A-:B-]					
SAMN31968666	[F2:A-:B-]					
SAMN31968667	[F24:A-:B6]	+				+
SAMN31968668	[F24:A-:B1], IncI1/ST3 CC3	+	+	+	+	+
SAMN31968669	[F18:A-:B1], IncHI2/ST4	+	+	+	+	+
SAMN31968670	[F18:A-:B1], IncHI2/ST4	+	+	+	+	+
SAMN31968671	[F18:A-:B1], IncI1/ST3 CC3	+			+	+
SAMN31968672	[F18:A-:B8]	+			+	+
SAMN31968673	[F18:A-:B1]	+	+	+	+	+
SAMN31968674	[F18:A6:B8]	+				
SAMN31968675	[F24:A-:B6]	+				+
SAMN31968676	[F16:A-:B-]	+				+
SAMN31968677	[F-:A-:B1]	+				+
SAMN31968678	[F-:A-:B1]					+
SAMN31968679	[F18:A-:B1], IncI1/ST25 CC2	+	+	+	+	+
SAMN31968680	[F18:A-:B1], IncI1/ST25 CC2	+	+	+	+	+
SAMN31968681	[F24:A-:B1]	+				+
SAMN31968682	[F16:A-:B1]					
SAMN31968685	[F2:A-:B1]	+	+	+	+	+
SAMN31968686	[F2:A-:B1]	+	+	+	+	+
SAMN31968687	IncI1/ST3 CC3					
SAMN31968688	IncI1/ST3 CC3					
SAMN31968689	[F18:A-:B1]	+	+	+	+	+
SAMN31968690	[F40:A-:B-]					
SAMN31968691	[F16:A-:B-]					
SAMN31968692	[F57:A-:B42]					
SAMN31968693	[F18:A-:B1]	+				+
SAMN31968694	[F18:A-:B1]	+				+
SAMN31968695	[F18:A-:B6], IncI1/ST3 CC3					
SAMN31968696	[F16:A-:B1]	+				
SAMN31968697	[F24:A-:B1]	+	+	+	+	+
SAMN31968698	[F24:A-:B1]	+	+	+	+	+
SAMN31968699	[F24:A-:B1], IncHI2/ST4	+			+	+
SAMN31968700	[F18:A-:B-], IncI1/ST3 CC3	+	+	+	+	+
SAMN31968701	[F18:A-:B1]	+	+	+	+	+
SAMN31968702	[F18:A6:B8]	+				
SAMN31968703	[F18:A-:B1]	+				+
SAMN31968704	[F18:A-:B8], IncHI2/ST2	+	+	+	+	+
SAMN31968705	[F18:A6:B1], IncHI2/ST4	+				+
SAMN31968706	[F18:A-:B1]	+				+
SAMN31968707	IncHI2/ST3					
SAMN31968708	[F-:A-:B-]	+	+	+		+
SAMN31968709	[F24:A-:B1]	+			+	+
SAMN31968710	[F16:A-:B1]	+				+
SAMN31968711	[F46:A-:B20]					
SAMN31968712	[F46:A-:B20]					
SAMN31968713	[F-:A-:B1]	+	+	+	+	+
SAMN31968714	[F18:A-:B1], IncI1/ST36 CC3	+				
SAMN31968715	[F18:A6:B8]	+				
SAMN31968716	[F18:A6:B8]	+				
SAMN31968717	[F18:A6:B8]	+				
SAMN31968718	[F-:A-:B20], IncI1/ST7 CC7	+	+	+		+
SAMN31968719	[F24:A-:B1]	+			+	+
SAMN31968720	[F24:A-:B1]	+			+	+
SAMN31968721	[F24:A-:B1]	+				+
SAMN31968722	[F24:A-:B1]	+				
SAMN31968723	[F18:A-:B1]	+				+
SAMN31968724	[F36:A6:B1], IncI1/ST214* CC2	+	+	+	+	+
SAMN31968725	[F18:A5:B1]	+	+	+	+	+
SAMN31968726	[F18:A5:B1]	+	+	+	+	+
SAMN31968727	[F18:A-:B27]	+			+	+
SAMN31968728	[F18:A-:B27]	+			+	+
SAMN31968729	[F24:A-:B6]	+				+
SAMN31968730	[F24:A-:B1], IncHI2/ST4	+	+	+	+	+
SAMN31968731	[F24:A-:B1], IncHI2/ST4	+	+	+	+	+
SAMN31968732	[F40:A-:B-]					
SAMN31968733	[F40:A-:B-]					
SAMN31968734	[F57:A-:B42]					
SAMN31968735	[F-:A-:B1]	+	+	+	+	+
SAMN31968736	[F18:A-:B1]	+				+
SAMN31968737	[F-:A-:B-]					
SAMN31968738	[F18:A6:B1]	+	+	+	+	+
SAMN31968739	[F18:A5:B1]	+	+	+	+	+
SAMN31968740	[F24:A-:B1]	+				+
SAMN31968741	[F24:A-:B1]	+				+
SAMN31968742	[F2:A-:B1]	+	+		+	+
SAMN31968743	[F18:A5:B1]	+	+	+	+	+
SAMN31968744	[F24:A-:B1]					+
SAMN31968745	[F18:A6:B8], IncHI2/ST4	+				
SAMN31968746	[F18:A-:B1]	+	+	+	+	+
SAMN31968747	[F18:A-:B1]	+	+	+	+	+
SAMN31968748	[F18:A-:B1]	+				+
SAMN31968749	IncI1/ST3 CC3					
SAMN31968750	[F35:A-:B-]					
SAMN31968751	[F10:A-:B1]	+	+	+	+	+

a: MCA-MacConkey agar; EMB-Methylene Blue Agar

b: CZ-Czech Republic; DE-Germany; PL-Poland; UKR-Ukraine

c: MLST-multilocus sequence typing; ST-sequence type

d: nt-not tested

e: COL-colistin; AMP-ampicillin; S-streptomycin; S3-sulphonamides; TE-tetracycline; SXT-trimethoprim/sulfamethoxazole; C-chloramphenicol; KZ-cefazolin; NA-nalidix acid; CAZ-ceftazidime; CN-gentamicin; AMC-amoxicillin/clavulanate; CIP-ciprofloxacin; FOS-fosfomycine; ETP-ertapenem; IMP-imipenem; MER-meropenem; ATM-azithromycin; F-mitorfuranzoin; AZM-aztreonam; TGC-tigecycline

f: Lin-lincosamide; Qui-quinilone; Suplhonam-suplhonamide

g: pMLST-plasmid multilocus sequence typing; CC-clonal complex

Antibiotic resistance profile

Resistant

Intermediate

Susceptible

Antibiotic resistance genes: min. 95% length coverage and min. 95% identity to the reference sequence

Chromosomal point mutations conferring with antimicrobial resistance: gyrA, parC, parE for mutations associated with quinolones, fluoroquinolones; pmrA, pmrB for mutations associated with colistin resistance.

Plasmids replicons: min. 90% length coverage and min. 90% identity to the reference sequence

Table S2: Comparative analysis of antibiotic susceptiblity testing and detection of resistance via WGS of *mcr-* carrying *E. coli* isolates

	Phenotypic resistance summary N=111						Comparison of phenotypic resistance with expected restance (based on WGS) *N=109				
	Resistant		Intermediate		Susceptible		GM without resitance	GM absence	Discordance		Corcordance
	n	%	n	%	n	%	n	n	n	%	%
**AMP**	111	100,00	0	0,00	0	0,00	0	3	3	2,70	97,30
**S**	93	83,78	18	16,22	0	0,00	1	24	25	22,52	77,48
**S3**	90	81,08	5	4,50	17	15,32	0	2	2	1,80	98,20
**TE**	96	86,49	0	0,00	16	14,41	0	2	2	1,80	98,20
**SXT**	80	72,07	0	0,00	32	28,83	1	2	3	2,70	97,30
**C**	50	45,05	2	1,80	60	54,05	0	9	9	8,11	91,89
**KZ**	34	30,63	0	0,00	78	70,27	15	1	16	14,41	85,59
**NA**	96	86,49	6	5,41	10	9,01	0	3	3	2,70	97,30
**CAZ**	5	4,50	7	6,31	100	90,09	18	0	18	16,22	83,78
**CN**	19	17,12	9	8,11	84	75,68	0	3	3	2,70	97,30
**AMC**	26	23,42	57	51,35	29	26,13	0	2	2	1,80	98,20
**CIP**	79	71,17	15	13,51	18	16,22	8	3	11	9,91	90,09
**ETP**	0	0,00	1	0,90	111	100,00	0	0	0	0,00	100,00
**MER**	1	0,90	2	1,80	109	98,20	0	1	1	0,90	99,10
**IPM**	1	0,90	8	7,21	103	92,79	0	1	1	0,90	99,10
**AZM**	27	24,32	0	0,00	85	76,58	17	0	17	15,32	84,68
**COL**	111	100,00	0	0,00	0	0,00	ND	ND	ND	ND	ND
**FOS**	1	0,90	0	0,00	111	100,00	0	1	1	0,90	99,10
**TGC**	0	0,00	0	0,00	111	100,00	0	0	0	0,00	100,00
**ATM**	10	9,01	1	0,90	101	90,99	4	18	22	19,82	80,18
**F**	7	6,31	33	29,73	72	64,86	ND	ND	ND	ND	ND

Legend

ND - not determined

*2 isolates were excluded due to low quality of WGS data

AMP: ampicillin

S: streptomycin

S3: sulphonamides

TE: tetracycline

SXT: trimethoprim/sulfamethoxazole

C: chloramphenicol

KZ: cefazolin

NA: nalidix acid

CAZ: ceftazidime

CN: gentamicin

AMC: amoxicillin/clavulanate

CIP: ciprofloxacin

ETP: ertapenem

MER: meropenem

IMP: imipenem

AZM: aztreonam

COL: colistin

FOS: fosfomycine

TGC: tigecycline

ATM: azithromycin

F: nitrofurantoin

Table S3: SNP analysis of selected *E. coli* STs

ST69

Isolates	50774	54076	279_EMB
50774	0	9456	4352
54076	9456	0	8758
279_EMB	4352	8758	0

ST88

Isolates	734_MCA	734_EMB	48964-19	57467	60377
734_MCA	0	1	6733	9345	9541
734_EMB	1	0	6734	9344	9540
48964-19	6733	6734	0	10379	10082
57467	9345	9344	10379	0	2825
60377	9541	9540	10082	2825	0

ST131

Isolates	6645_MCA	6645_EMB	54164
6645_MCA	0	48	1488
6645_EMB	48	0	1430
54164	1488	1430	0

ST162

Isolates	2049_MCA	2049_EMB	5417_MCA	5417_EMB	5533_MCA	7065_MCA	60233	60461	7506_EMB	7674_MCA	8564_MCA	8564_EMB
2049_MCA	0	5	2031	2034	1953	1810	2803	2807	1922	2038	2108	2110
2049_EMB	5	0	2033	2036	1958	1815	2808	2812	1927	2043	2110	2112
5417_MCA	2031	2033	0	0	2379	2026	2561	2563	2184	3141	2545	2549
5417_EMB	2034	2036	0	0	2382	2026	2561	2563	2184	3144	2545	2549
5533_MCA	1953	1958	2379	2382	0	1906	2968	2966	2174	2849	2478	2481
7065_MCA	1810	1815	2026	2026	1906	0	2618	2621	1885	2789	2235	2240
60233	2803	2808	2561	2561	2968	2618	0	50	2665	3749	3180	3181
60461	2807	2812	2563	2563	2966	2621	50	0	2668	3755	3183	3184
7506_EMB	1922	1927	2184	2184	2174	1885	2665	2668	0	2923	2448	2454
7674_MCA	2038	2043	3141	3144	2849	2789	3749	3755	2923	0	3181	3183
8564_MCA	2108	2110	2545	2545	2478	2235	3180	3183	2448	3181	0	19
8564_EMB	2110	2112	2549	2549	2481	2240	3181	3184	2454	3183	19	0

ST744

Isolates	5501_EMB	45082	48907	52637	52857	54343	54444-19	55923-3-20	58559	60061	60462	7674_EMB	7727_MCA	7727_EMB	7837_EMB	8211_EMB	4041_MCA
5501_EMB	0	284	1307	110	1324	1314	399	1329	1443	1414	2372	1373	1289	1345	1289	1303	1327
45082	284	0	1383	333	1397	1387	237	1402	1484	1470	2449	1436	1362	1365	1362	1301	1402
48907	1307	1383	0	1356	107	114	1500	112	683	853	1439	817	72	572	73	906	112
52637	110	333	1356	0	1366	1344	451	1359	1490	1456	2423	1410	1332	1392	1332	1348	1371
52857	1324	1397	107	1366	0	78	1513	75	697	863	1456	829	82	584	82	923	65
54343	1314	1387	114	1344	78	0	1502	9	705	849	1452	805	90	590	90	913	83
54444-19	399	237	1500	451	1513	1502	0	1518	1599	1585	2564	1551	1477	1467	1478	1416	1518
55923-3-20	1329	1402	112	1359	75	9	1518	0	702	865	1461	821	87	589	87	928	80
58559	1443	1484	683	1490	697	705	1599	702	0	1046	1530	1008	662	874	662	1151	700
60061	1414	1470	853	1456	863	849	1585	865	1046	0	1818	229	828	825	828	1025	868
60462	2372	2449	1439	2423	1456	1452	2564	1461	1530	1818	0	1779	1421	1678	1421	1975	1461
7674_EMB	1373	1436	817	1410	829	805	1551	821	1008	229	1779	0	791	789	791	986	832
7727_MCA	1289	1362	72	1332	82	90	1477	87	662	828	1421	791	0	549	0	888	87
7727_EMB	1345	1365	572	1392	584	590	1467	589	874	825	1678	789	549	0	549	894	587
7837_EMB	1289	1362	73	1332	82	90	1478	87	662	828	1421	791	0	549	0	888	87
8211_EMB	1303	1301	906	1348	923	913	1416	928	1151	1025	1975	986	888	894	888	0	926
4041_MCA	1327	1402	112	1371	65	83	1518	80	700	868	1461	832	87	587	87	926	0

ST1011

Isolates	1357_MCA	BA397-4_MC	4282_EMB	4640_MCA	4640_EMB	4804_EMB	5391_EMB	5674_MCA	5674_EMB	52230	54345	56354	7410_MCA	7410_EMB	7506_MCA	8799_MCA	8799_EMB	8950_MCA	8950_EMB
1357_MCA	0	2193	2283	3013	2979	2338	2282	388	387	3135	2529	2212	2278	2249	2861	2295	2299	2237	2238
BA397-4_MC	2193	0	2536	3576	3542	2726	2535	2378	2377	3762	3005	38	2531	2486	1595	3107	3111	54	54
4282_EMB	2283	2536	0	2373	2339	1621	1	2277	2276	2669	1672	2566	2	18	2176	2038	2042	2576	2577
4640_MCA	3013	3576	2373	0	34	2337	2374	3169	3168	1501	2257	3606	2368	2341	2676	1134	1138	3580	3581
4640_EMB	2979	3542	2339	34	0	2303	2340	3135	3134	1457	2227	3570	2334	2307	2642	1100	1104	3555	3547
4804_EMB	2338	2726	1621	2337	2303	0	1620	2500	2499	2431	1780	2745	1621	1587	1969	1821	1825	2734	2735
5391_EMB	2282	2535	1	2374	2340	1620	0	2276	2275	2661	1660	2555	1	17	2175	2037	2041	2575	2576
5674_MCA	388	2378	2277	3169	3135	2500	2276	0	1	3332	2292	2398	2272	2231	3059	2455	2459	2382	2383
5674_EMB	387	2377	2276	3168	3134	2499	2275	1	0	3331	2291	2397	2271	2230	3058	2454	2458	2381	2382
52230	3135	3762	2669	1501	1457	2431	2661	3332	3331	0	2415	3768	2654	2616	3025	1541	1533	3816	3806
54345	2529	3005	1672	2257	2227	1780	1660	2292	2291	2415	0	3012	1656	1606	2629	1912	1904	3052	3019
56354	2212	38	2566	3606	3570	2745	2555	2398	2397	3768	3012	0	2551	2493	1607	3126	3118	101	83
7410_MCA	2278	2531	2	2368	2334	1621	1	2272	2271	2654	1656	2551	0	18	2171	2033	2037	2571	2572
7410_EMB	2249	2486	18	2341	2307	1587	17	2231	2230	2616	1606	2493	18	0	2142	2004	1996	2526	2527
7506_MCA	2861	1595	2176	2676	2642	1969	2175	3059	3058	3025	2629	1607	2171	2142	0	2351	2355	1639	1639
8799_MCA	2295	3107	2038	1134	1100	1821	2037	2455	2454	1541	1912	3126	2033	2004	2351	0	8	3111	3113
8799_EMB	2299	3111	2042	1138	1104	1825	2041	2459	2458	1533	1904	3118	2037	1996	2355	8	0	3116	3117
8950_MCA	2237	54	2576	3580	3555	2734	2575	2382	2381	3816	3052	101	2571	2526	1639	3111	3116	0	1
8950_EMB	2238	54	2577	3581	3547	2735	2576	2383	2382	3806	3019	83	2572	2527	1639	3113	3117	1	0

Legend
Isolates from this study
Isolates from humans (Zelendova et al. 2023, review in Frontiers in Microbiology)

Table S4. Primers used for PCR detection of *mcr* genes

Primer	Sequence (5'-3')	Target gene	Reference	Type of reaction	Size (bp)
CLR_F	CGGTCAGTCCGTTTGTTC	*mcr-1*	(24)	simplex	309
CLR_R	CTTGGTCGGTCTGTAGGG				
mcr-2_F	TGTTGCTTGTGCCGATTGGA	*mcr-2*	(25)	simplex	566
mcr-2_R	AGATGGTATTGTTGGTTGCTG				
mcr-3_F	TTGGCACTGTATTTTGCATTT	*mcr-3*	(26)	simplex	542
mcr-3_R	TTAACGAAATTGGCTGGAACA				
mcr-4_F	ATTGGGATAGTCGCCTTTTT	*mcr-4*	(27)	simplex	487
mcr-4_R	TTACAGCCAGAATCATTATCA				
mcr-5_F	GGTTGAGCGGCTATGAAC	*mcr-5*	(28)	multiplex 1*	205
mcr-5_R	GAATGTTGACGTCACTACGG				
mcr-6_F	GTCCGGTCAATCCCTATCTGT	*mcr-6*	(29)	multiplex 2**	556
mcr-6_R	ATCACGGGATTGACATAGCTAC				
mcr-7_F	GGCGACCTCCTACCTGAATG	*mcr-7*	This study***		333
mcr-7_R	ACTTTGGCATCAGTCCCCAG				
mcr-8_F	AACCGCCAGAGCACAGAATT	*mcr-8*	(30)		667
mcr-8_R	TTCCCCCAGCGATTCTCCAT				
mcr-9_F	CGGTACCGCTACCGCAATAT	*mcr-9*	(31)	multiplex 1*	131
mcr-9_R	ATAACAGCGAGACACCGGTT				
mcr-10_F	GGACCGACCTATTACCAGCG	*mcr-10*	(32)	simplex	366
mcr-10_R	GGCATTATGCTGCAGACACG				

*** multiplex reaction of *mcr-5*, *mcr-9***
**** multiplex reaction of *mcr-6*, *mcr-7*, *mcr-8***
***** The primers were designed using *mcr-7.1* variant recovered from GenBank** (mcr7.1_1_MG267386)

PCR detection program: 1. 94°C 10min, 2. 94°C 1 min, 3. 60 °C 1 min (TA), 4. 72°C 1 min (step 2-4 30x repeat), 5. 72 °C 5 min, 6. 10°C/ ∞

Table S5: Assembly statistics.

Sample	Medium	BioSample	Assembly name	Contigs >= 0bp	Contigs >1000bp	Contigs >5000bp	Contigs >10000bp	Contigs >25000bp	Contigs >50000bp	Total lenght >= 0bp	Total length >1000bp	Total length >5000bp	Total length >10000bp	Total length >25000bp	Total length >50000bp	Contigs	Largest contig	Total length	GC (%)	N50	N90	auN	L50	L90	# N's per 100 kbp
3180/1	MCA	SAMN31968641	3180_1_MCA	147	105	76	64	53	38	5058949	5040295	4978181	4899782	4688686	4173975	125	295862	5052939	50.49	107362	30579	115924.9	17	48	0.00
3180/1	EMB	SAMN31968642	3180_1_EMB	149	105	77	65	53	38	5060305	5040486	4981381	4902982	4672706	4148890	127	295862	5054203	50.48	107260	30579	115386.7	17	49	0.00
3286	MCA	SAMN31968643	3286_MCA	191	116	83	71	48	33	5053409	5019813	4939428	4852183	4476905	3952408	144	488121	5038160	50.52	103602	23094	152469.7	14	51	0.00
3286	EMB	SAMN31968644	3286_EMB	167	112	75	60	45	30	4720836	4699148	4622009	4504554	4225048	3658593	128	323741	4709479	50.62	96536	23631	139003.8	13	46	0.00
3423	EMB	SAMN31968645	3423_EMB	171	114	72	57	42	29	4922169	4896317	4802589	4695752	4471573	4017833	140	378040	4913374	50.46	133412	27056	164719.3	11	41	0.00
3845	MCA	SAMN31968646	3845_MCA	126	95	67	57	44	26	5134323	5118161	5046650	4976369	4745976	4041236	111	520931	5129682	50.54	127655	34931	191250.4	10	40	0.00
3845	EMB	SAMN31968647	3845_EMB	223	104	75	64	48	31	5325774	5285871	5216961	5138953	4893434	4284034	128	349621	5301893	50.32	122709	29667	147485.1	14	44	0.00
4041	MCA	SAMN31968648	4041_MCA	154	108	78	64	50	31	4972479	4951991	4886070	4783336	4559339	3864221	123	407410	4962428	50.54	106460	29387	148270.1	13	47	0.00
4067	MCA	SAMN31968649	4067_MCA	126	84	62	55	40	26	5032220	5014323	4959725	4907953	4642900	4149354	97	411422	5024227	50.48	175849	27792	186106.5	10	36	0.00
4067	EMB	SAMN31968650	4067_EMB	145	92	63	55	39	26	5080298	5054904	4985158	4923960	4658743	4215386	115	401493	5071346	50.47	175849	26934	179152.6	10	36	0.00
4144	MCA	SAMN31968651	4144_MCA	134	84	57	49	39	28	4914862	4892346	4822558	4762951	4578087	4204443	105	357709	4906633	50.55	173781	31342	171204.7	10	34	0.00
4144	EMB	SAMN31968652	4144_EMB	182	77	58	54	43	27	5391763	5352871	5317375	5289171	5102826	4523494	99	500716	5368222	50.49	198590	33361	212070.9	9	35	0.00
4281	EMB	SAMN31968653	4281_EMB	180	110	72	65	52	34	5164382	5134349	5038381	4991507	4783126	4119595	133	331013	5150671	50.33	113156	29718	131540.6	15	47	0.00
4282	EMB	SAMN31968654	4282_EMB	119	74	48	42	34	23	5308464	5289360	5225561	5183821	5047753	4673771	91	704078	5300865	50.38	223804	41184	320783.3	6	26	0.00
4526	MCA	SAMN31968655	4526_MCA	149	97	65	53	42	30	4990093	4966706	4887850	4807991	4613274	4172158	119	483746	4982091	50.68	134994	32688	173479.8	11	38	0.00
4526	EMB	SAMN31968656	4526_EMB	160	103	63	54	43	28	4998403	4971179	4877914	4815535	4634046	4063863	131	483744	4990083	50.68	154762	32688	188693.0	10	39	0.00
4633	MCA	SAMN31968657	4633_MCA	68	53	37	35	31	22	4874487	4868356	4826519	4810501	4755113	4442957	57	615931	4870705	50.48	280780	57825	296325.8	6	21	0.00
4633	EMB	SAMN31968658	4633_EMB	108	71	49	44	37	27	5129180	5114320	5068514	5027707	4899596	4517353	82	531023	5122062	50.52	173407	46522	222035.5	9	29	0.00
4640	MCA	SAMN31968659	4640_MCA	149	89	49	41	35	23	5173691	5144718	5046183	4987186	4882200	4479563	117	816487	5163199	50.52	325036	33316	316411.5	6	28	0.00
4640	EMB	SAMN31968660	4640_EMB	150	89	48	40	34	23	5174490	5145156	5050408	4987831	4873984	4496363	116	819247	5163074	50.52	325036	35333	319492.1	6	27	0.00
4689	EMB	SAMN31968661	4689_EMB	188	111	76	63	49	36	5457811	5428354	5336910	5242326	5000747	4580406	133	354841	5442672	50.25	140495	26349	149880.6	13	46	0.00
4804	EMB	SAMN31968662	4804_EMB	178	99	63	54	40	24	5327194	5287206	5203239	5144162	4939969	4343503	136	680913	5313372	50.40	203423	33147	236415.3	9	35	0.00
5027	MCA	SAMN31968663	5027_MCA	130	87	68	62	49	34	5391278	5371831	5331970	5291623	5092363	4554539	105	604257	5383776	50.37	119982	35273	180446.9	13	42	0.00
5027	EMB	SAMN31968664	5027_EMB	192	125	85	69	51	30	5398933	5369589	5263383	5147537	4857078	4140215	149	393791	5385725	50.32	119533	25016	150756.5	13	51	0.00
5028	MCA	SAMN31968665	5028_MCA	228	109	83	68	50	34	5124887	5085224	5020467	4912326	4623392	4058267	126	312020	5097154	50.45	107040	28838	126756.0	15	49	0.00
5028	EMB	SAMN31968666	5028_EMB	132	100	69	56	45	29	5122058	5109386	5028147	4932163	4738662	4167682	109	578019	5115453	50.46	122983	30955	207450.6	10	41	0.00
5391	MCA	SAMN31968667	5391_MCA	264	122	82	65	46	27	5140796	5092541	5011312	4897159	4586980	3943315	147	410105	5110261	50.49	135647	24201	163242.0	12	47	0.00
5391	EMB	SAMN31968668	5391_EMB	137	81	51	45	36	24	5410489	5387412	5316969	5275227	5112576	4689665	100	704078	5400001	50.37	223804	39602	300851.4	7	28	0.00
5417	MCA	SAMN31968669	5417_MCA	119	73	52	46	36	27	5279636	5255617	5211895	5169782	5001476	4694286	97	586045	5272492	50.35	184942	43775	237929.0	8	29	0.00
5417	EMB	SAMN31968670	5417_EMB	121	75	54	48	38	28	5279992	5255972	5212250	5170137	5001831	4668584	99	586167	5272847	50.35	167145	43775	229443.4	9	30	0.00
5501	MCA	SAMN31968671	5501_MCA	138	110	82	77	58	37	5021794	5007479	4940386	4903675	4589726	3822478	125	237143	5017886	50.56	93706	27779	100727.8	19	56	0.00
5501	EMB	SAMN31968672	5501_EMB	151	103	70	63	48	30	4879127	4858920	4789269	4732876	4501220	3867845	119	347954	4870364	50.47	119243	30276	145935.3	12	44	0.00
5533	MCA	SAMN31968673	5533_MCA	120	70	53	46	36	26	5047289	5022867	4979888	4927948	4748628	4428361	92	625534	5038859	50.57	193163	35000	279579.2	7	29	0.00
5533	EMB	SAMN31968674	5533_EMB	132	99	65	57	44	31	5053394	5037415	4953834	4893394	4644769	4194770	113	327204	5047898	50.49	147012	30089	145396.1	13	41	0.00
5568/2	MCA	SAMN31968675	5568_2_MCA	252	125	99	85	54	26	4973309	4929384	4869546	4773978	4248675	3266091	147	256864	4943844	50.66	92245	19254	113880.7	15	63	0.00
5568/2	EMB	SAMN31968676	5568_2_EMB	202	122	71	61	47	34	5028788	4985264	4865213	4789761	4565665	4125085	173	285037	5020001	50.68	113369	26055	129086.2	14	46	0.00
5674	MCA	SAMN31968677	5674_MCA	93	51	34	29	25	17	5030678	5008481	4973123	4930112	4852720	4568327	76	942048	5025382	50.41	343857	69726	403513.2	5	17	0.00
5674	EMB	SAMN31968678	5674_EMB	91	52	33	30	26	18	5031467	5009680	4973264	4950118	4883352	4607804	78	981523	5027290	50.41	291445	69726	412003.9	5	17	0.00
6029	MCA	SAMN31968679	6029_MCA	171	122	91	78	55	33	5112702	5091592	5018348	4913128	4521192	3775367	138	304486	5102204	50.54	102624	22022	118532.6	16	59	0.00
6029	EMB	SAMN31968680	6029_EMB	179	124	90	79	56	31	5112181	5087969	5004218	4919806	4527274	3664618	145	304486	5101385	50.54	92307	22292	121988.1	15	59	0.00
6031	EMB	SAMN31968681	6031_EMB	278	145	81	65	42	28	5218866	5155781	5034733	4918628	4553043	4044009	202	408872	5194179	50.52	137150	19100	164880.2	12	48	0.00
6406	EMB	SAMN31968682	6406_EMB	126	91	68	55	40	28	4929956	4916248	4864362	4767798	4508015	4048751	100	362887	4922242	50.54	128407	32767	171002.2	11	38	0.00
6645	MCA	SAMN31968685	6645_MCA	69	49	34	29	27	20	5113606	5102472	5067843	5037476	5000258	4767931	62	718492	5111468	50.58	340541	68330	379391.4	5	18	0.00
6645	EMB	SAMN31968686	6645_EMB	73	50	35	30	27	20	5114209	5102043	5067505	5037138	4983106	4750779	63	718523	5111039	50.57	340541	68330	378826.3	5	18	0.00
6646	MCA	SAMN31968687	6646_MCA	143	96	67	58	47	35	4937205	4917371	4854694	4796042	4611470	4205688	109	358266	4926482	50.81	122954	31071	145214.2	14	41	0.00
6646	EMB	SAMN31968688	6646_EMB	147	98	67	59	47	35	4929378	4906667	4838169	4786513	4584608	4186396	117	358472	4919987	50.81	126494	30242	136565.9	14	42	0.00
7065	MCA	SAMN31968689	7065_MCA	143	91	62	54	44	30	5189523	5165439	5096604	5040330	4886400	4387141	110	594212	5178964	50.44	150626	34982	189558.7	11	37	0.00
7065	EMB	SAMN31968690	7065_EMB	113	76	55	51	43	30	5137020	5122076	5078034	5049029	4913285	4432047	88	326058	5130139	50.46	149913	40333	166745.6	11	35	0.00
7168	MCA	SAMN31968691	7168_MCA	141	88	61	48	39	30	5097525	5068336	5008713	4907694	4753220	4420891	122	540319	5091010	50.47	148032	40750	185190.3	11	34	0.00
7168	EMB	SAMN31968692	7168_EMB	114	95	71	62	48	30	4803995	4795885	4734852	4671799	4437786	3754031	101	336450	4799440	50.56	121115	30145	132722.4	14	44	0.00
7208	MCA	SAMN31968693	7208_MCA	229	129	73	57	42	27	5299885	5249763	5124476	5006805	4764278	4242961	172	842181	5281461	50.41	167146	25452	252337.5	9	42	0.00
7208	EMB	SAMN31968694	7208_EMB	187	111	73	60	42	28	5237670	5203700	5114529	5020527	4746607	4252600	137	528512	5223117	50.41	167146	26300	214590.6	9	41	0.00
7335	MCA	SAMN31968695	7335_MCA	158	108	78	66	50	32	4998414	4976273	4907939	4820251	4547264	3913728	129	400611	4990081	50.71	111979	28229	136417.7	14	48	0.00
7335	EMB	SAMN31968696	7335_EMB	128	88	66	58	48	34	4983732	4963706	4910063	4847484	4711524	4227215	108	380008	4978264	50.68	113373	37057	153834.5	13	41	0.00
7410	MCA	SAMN31968697	7410_MCA	133	86	59	50	38	23	5432275	5411940	5348934	5285494	5075094	4546531	102	704078	5422355	50.38	230143	33776	272631.4	7	32	0.00
7410	EMB	SAMN31968698	7410_EMB	156	95	59	49	39	24	5568794	5541507	5457239	5392623	5218023	4697099	120	704078	5558075	50.33	221478	33776	292700.6	7	32	0.00
7506	MCA	SAMN31968699	7506_MCA	147	93	56	53	35	23	5369239	5344265	5259968	5235604	4951349	4534699	114	766783	5358699	50.32	223804	31816	290655.8	7	31	0.00
7506	EMB	SAMN31968700	7506_EMB	125	78	60	52	41	29	5077692	5056282	5018266	4958630	4761148	4340667	96	582070	5068681	50.58	149887	34982	200703.7	10	35	0.00
7674	MCA	SAMN31968701	7674_MCA	154	87	61	56	44	28	5255515	5225902	5163780	5128312	4944266	4368508	111	416370	5243871	50.35	185062	33159	199375.4	9	37	0.00
7674	EMB	SAMN31968702	7674_EMB	149	91	68	58	44	29	4811538	4794743	4746052	4664655	4428543	3905155	98	386644	4799758	50.55	120990	30545	163558.2	11	40	0.00
7727	MCA	SAMN31968703	7727_MCA	151	85	61	55	41	27	4745374	4724798	4671864	4623274	4391581	3905319	96	407425	4732439	50.52	127892	30955	176732.3	10	37	0.00
7727	EMB	SAMN31968704	7727_EMB	223	127	88	68	49	29	5238889	5200636	5118334	4971487	4646457	3920933	154	416751	5220305	50.29	119243	23624	156004.4	12	52	0.00
7837	MCA	SAMN31968705	7837_MCA	209	129	94	83	56	35	5228817	5197802	5118575	5040123	4610375	3859724	150	323993	5213311	50.38	95545	23082	116619.1	17	60	0.00
7837	EMB	SAMN31968706	7837_EMB	134	83	64	60	46	31	4743482	4727015	4684571	4652765	4429193	3876368	93	407425	4734066	50.52	119249	31950	151605.2	12	41	0.00
8211	MCA	SAMN31968707	8211_MCA	114	61	48	43	40	33	5288173	5266808	5237832	5203509	5145558	4899687	82	417288	5280698	50.18	193168	62458	202682.9	9	31	0.00
8211	EMB	SAMN31968708	8211_EMB	173	109	72	63	44	30	4920472	4896475	4812341	4752382	4450917	3957146	124	381065	4906934	50.58	125687	25328	155510.7	12	43	0.00
8482	MCA	SAMN31968709	8482_MCA	220	116	79	65	46	35	5135560	5099354	5015368	4908438	4565945	4142899	148	310110	5120679	50.41	121472	23478	123272.4	15	48	0.00
8482	EMB	SAMN31968710	8482_EMB	148	90	67	59	47	34	5001037	4979005	4925121	4862704	4696822	4250035	108	380008	4991919	50.67	111922	33291	153955.3	13	40	0.00
8563	MCA	SAMN31968711	8563_MCA	201	110	81	71	48	32	4977120	4945464	4878786	4812999	4420976	3812562	134	358999	4962173	50.59	114982	23104	132838.7	14	50	0.00
8563	EMB	SAMN31968712	8563_EMB	215	125	82	74	49	33	5077305	5047613	4938028	4883162	4467486	3881885	147	358999	5062867	50.58	112372	22573	131643.0	15	53	0.00
8564	MCA	SAMN31968713	8564_MCA	186	76	52	44	37	26	5053681	5012301	4965104	4902490	4776637	4417537	106	630783	5033865	50.51	187173	41298	256123.4	8	29	0.00
8564	EMB	SAMN31968714	8564_EMB	180	79	53	45	39	29	5204936	5168584	5112707	5051450	4951672	4627118	104	630783	5186238	50.46	187173	46949	261093.5	8	30	0.00
8615	MCA	SAMN31968715	8615_MCA	179	111	86	72	53	34	5011024	4986126	4932804	4820792	4510714	3830862	132	396169	4999977	50.42	104580	25887	126236.6	15	53	0.00
8615	EMB	SAMN31968716	8615_EMB	185	113	88	73	54	33	5014450	4986620	4930580	4810924	4497148	3751079	138	396169	5003120	50.42	104580	24588	125260.4	15	55	0.00
8622	MCA	SAMN31968717	8622_MCA	177	116	92	74	53	35	5063590	5037844	4985808	4842945	4491973	3836671	140	396169	5053806	50.38	98258	23103	123183.3	16	56	0.00
8693	EMB	SAMN31968718	8693_EMB	183	116	73	62	47	34	5281852	5259003	5169078	5085320	4819968	4351258	131	567675	5269241	50.55	126127	29327	173855.4	12	45	0.00
8799	MCA	SAMN31968719	8799_MCA	202	72	46	42	30	21	5234942	5200894	5139953	5104465	4899699	4595234	92	727224	5214900	50.41	309216	38928	306573.3	6	24	0.00
8799	EMB	SAMN31968720	8799_EMB	144	76	47	43	31	22	5227149	5203833	5138561	5103073	4898307	4596762	94	727224	5216150	50.41	309216	38928	302586.6	6	25	0.00
8815	MCA	SAMN31968721	8815_MCA	97	56	35	33	28	21	4991662	4972269	4923203	4908801	4823367	4597538	77	699833	4985540	50.57	362484	59393	355137.9	5	19	0.00
8815	EMB	SAMN31968722	8815_EMB	344	161	90	71	47	31	5361491	5291415	5133535	4996573	4598449	4021919	218	408938	5330570	50.53	111740	17066	143080.4	14	57	0.00
8897	MCA	SAMN31968723	8897_MCA	174	104	70	58	42	26	5212043	5179832	5107904	5018854	4770996	4217645	131	615674	5199422	50.36	167146	28504	226850.7	9	39	0.00
8897	EMB	SAMN31968724	8897_EMB	202	146	112	86	56	33	5129404	5104468	5017998	4830245	4324728	3464524	168	489945	5119541	50.59	81632	17665	124165.8	19	70	0.00
8950	MCA	SAMN31968725	8950_MCA	111	73	49	41	30	18	5163539	5146002	5080762	5021930	4816971	4395450	88	579970	5156711	50.56	253880	33630	276776.5	7	24	0.00
8950	EMB	SAMN31968726	8950_EMB	118	75	50	40	28	19	5164980	5145022	5078572	5005698	4782646	4479722	93	627551	5157676	50.56	238757	32116	283771.0	7	24	0.00
8996	MCA	SAMN31968727	8996_MCA	118	75	62	59	46	32	4979083	4957858	4933081	4912687	4720230	4221023	97	326318	4973078	50.61	139602	37691	144150.1	13	38	0.00
8996	EMB	SAMN31968728	8996_EMB	110	73	59	56	43	29	4976598	4957821	4930693	4910299	4717842	4220053	93	326318	4971642	50.61	151688	37691	160921.7	12	35	0.00
3959	EMB	SAMN31968729	3959_EMB	120	83	53	46	41	27	5047982	5033818	4982522	4934599	4844730	4373873	94	410919	5040494	50.70	173421	39084	188885.6	10	31	0.00
4063	MCA	SAMN31968730	4063_MCA	126	80	50	44	36	28	5219764	5197881	5134653	5089792	4967710	4659148	99	635647	5211657	50.20	210567	49759	233531.7	9	29	0.00
4063	EMB	SAMN31968731	4063_EMB	119	77	50	46	39	31	5169995	5150827	5090024	5058219	4943571	4627411	92	635647	5162299	50.24	167717	49760	209883.9	10	32	0.00
7066	MCA	SAMN31968732	7066_MCA	117	77	56	54	43	30	5138929	5122422	5079151	5062418	4894436	4432063	90	384488	5131464	50.47	149925	39768	169811.5	11	35	0.00
260	MCA	SAMN31968733	260_MCA	206	150	97	78	51	33	4865179	4837361	4713383	4569808	4110895	3466325	175	223726	4855702	50.64	92705	16283	94398.9	19	64	0.00
260	EMB	SAMN31968734	260_EMB	114	89	65	57	45	32	4816114	4806647	4745489	4690934	4494451	4027930	94	365689	4809630	50.55	125850	34273	145503.5	13	40	0.00
279	MCA	SAMN31968735	279_MCA	97	67	47	43	38	27	4770609	4754669	4705602	4675894	4593781	4187235	81	314532	4765043	50.55	181082	42080	180200.0	10	30	0.00
279	EMB	SAMN31968736	279_EMB	187	99	62	54	41	26	5173602	5132678	5058213	5002688	4809688	4341617	138	309964	5158657	50.50	204257	26667	173633.2	11	35	0.00
699	EMB	SAMN31968737	699_EMB	128	86	55	47	41	28	5055746	5034315	4972186	4917124	4815130	4322355	106	394876	5048589	50.62	171953	39751	190882.5	10	34	0.00
2008/1	MCA	SAMN31968738	2008_1_MCA	178	96	63	49	29	21	5584211	5548298	5474384	5370123	5021941	4748190	125	526920	5568106	50.30	281070	27057	265959.5	8	29	0.00
2049/2	MCA	SAMN31968739	2049_2_MCA	129	81	57	49	40	28	5120174	5098281	5031340	4975518	4806788	4403896	100	361421	5110989	50.56	177256	32706	191513.1	10	33	0.00
734	MCA	SAMN31968740	734_MCA	180	107	58	48	36	23	5159525	5121563	5010366	4940922	4729137	4228428	141	442967	5146933	50.57	198983	33125	210468.8	9	33	0.00
734	EMB	SAMN31968741	734_EMB	170	105	58	48	36	23	5158044	5122562	5017258	4947689	4735220	4234511	139	442760	5147755	50.57	188905	33125	213328.8	9	33	0.00
817	MCA	SAMN31968742	817_MCA	190	138	102	89	63	33	5100285	5074368	4983515	4892771	4414536	3337641	164	212938	5091718	50.53	81867	20256	87424.7	21	71	0.00
817	EMB	SAMN31968743	817_EMB	115	77	49	42	31	22	5325746	5306592	5237385	5190993	4981405	4659187	93	428235	5317626	50.39	244379	44406	243210.7	8	25	0.00
1357	MCA	SAMN31968744	1357_MCA	78	48	32	29	26	17	4910506	4897949	4852191	4828065	4776027	4457165	57	1139690	4904452	50.47	304061	58233	459500.6	5	17	0.00
1782/2	EMB	SAMN31968745	1782_2_EMB	206	128	93	78	54	32	5267798	5233846	5151986	5033094	4638690	3859213	157	423609	5253037	50.27	109385	20280	138157.5	15	59	0.00
2049/1	MCA	SAMN31968746	2049_1_MCA	141	86	56	46	41	27	5127401	5100817	5032298	4957513	4872879	4392939	108	632605	5116413	50.52	193392	38048	257746.1	7	32	0.00
2049/1	EMB	SAMN31968747	2049_1_EMB	143	85	55	45	40	26	5128173	5100803	5032263	4957478	4872843	4392923	107	632605	5116437	50.52	193392	38048	265757.8	7	31	0.00
2079/2	MCA	SAMN31968748	2079_2_MCA	167	100	59	48	34	23	5108121	5072648	4978029	4892419	4678366	4330685	133	537865	5095932	50.54	185521	26635	244378.5	8	31	0.00
BA 397/4	MCA	SAMN31968749	BA397-4_A_MCA	94	63	42	37	28	21	5117913	5102702	5056778	5022557	4869770	4638602	77	579870	5112302	50.51	396195	50190	310870.1	6	21	0.00
BA 1137/4	MCA	SAMN31968750	BA1137_4 _MCA	129	89	57	49	39	31	4882651	4865034	4783958	4730519	4586427	4355128	102	400692	4873384	50.61	129278	33537	176951.0	11	32	0.00
BA 1137/4	EMB	SAMN31968751	BA1137_4 _EMB	257	151	82	64	46	33	5033453	4980925	4815087	4692429	4402069	3938746	201	316559	5015213	50.75	111922	19362	121414.0	15	52	0.00
6407	EMB	excluded	6407_EMB	344	113	82	72	59	45	9021080	8962086	8889452	8820466	8615694	8143803	144	573610	8983954	45.38	226082	51341	238769.9	14	44	0.00
6407	MCA	excluded	6407_MCA	276	147	105	93	68	42	8931844	8890007	8783128	8691806	8272969	7409743	177	624333	8909344	45.28	223445	29290	228152.8	14	59	0.00

Supplementary References

1. Directive 2003/99/EC of the European Parliament and of the Council on the monitoring of zoonoses and zoonotic agents, amending Council Decision 90/424/EEC and repealing CouncilDirective 92/117/EEC. Official Journal L 325, 12 December 2003, pp. 31-40.

2. SVA Methodological Instruction No. 01/2014 laying down rules for regular microbiological examination of zoonotic agents carried out by the State 356/2004 Coll, on the surveillance (monitoring) of zoonoses and zoonotic agents.

3. CLSI. Performance Standards for Antimicrobial Susceptibility Testing. 27th ed. CLSI supplement M100. Wayne, PA: Clinical and Laboratory Standards Institute; 2017

4. Bolger AM, Lohse M, Usadel B. Trimmomatic: a flexible trimmer for Illumina sequence data. Bioinformatics. 2014 Aug 1;30(15):2114-20. doi: 10.1093/bioinformatics/btu170. Epub 2014 Apr 1. PMID: 24695404; PMCID: PMC4103590.

5. Bankevich A, Nurk S, Antipov D, Gurevich AA, Dvorkin M, Kulikov AS, Lesin VM, Nikolenko SI, Pham S, Prjibelski AD, Pyshkin AV, Sirotkin AV, Vyahhi N, Tesler G, Alekseyev MA, Pevzner PA. SPAdes: a new genome assembly algorithm and its applicationsto single-cell sequencing. J Comput Biol. 2012 May;19(5):455-77. doi: 10.1089/cmb.2012.0021. Epub 2012 Apr 16. PMID: 22506599; PMCID: PMC3342519.

6. Chen L, Zheng D, Liu B, Yang J, Jin Q. VFDB 2016: hierarchical and refined dataset for bigdata analysis--10 years on. Nucleic Acids Res. 2016 Jan 4;44(D1):D694-7. doi: 10.1093/nar/gkv1239. Epub 2015 Nov 17. PMID: 26578559; PMCID: PMC4702877.

7. Beghain J, Bridier-Nahmias A, Le Nagard H, Denamur E, Clermont O. ClermonTyping: aneasy-to-use and accurate in silico method for Escherichia genus strain phylotyping. MicrobGenom. 2018 Jul;4(7):e000192. doi: 10.1099/mgen.0.000192. Epub 2018 Jun 19. PMID: 29916797; PMCID: PMC6113867.

8. Siguier P, Perochon J, Lestrade L, Mahillon J, Chandler M. ISfinder: the reference centre forbacterial insertion sequences. Nucleic Acids Res. 2006 Jan 1;34(Database issue):D32-6. doi:10.1093/nar/gkj014. PMID: 16381877; PMCID: PMC1347377.

9. Alikhan NF, Petty NK, Ben Zakour NL, Beatson SA. BLAST Ring Image Generator (BRIG):simple prokaryote genome comparisons. BMC Genomics. 2011 Aug 8;12:402. doi: 10.1186/1471-2164-12-402. PMID: 21824423; PMCID: PMC3163573.

10. Wick RR, Judd LM, Gorrie CL, Holt KE. Unicycler: Resolving bacterial genome assemblies from short and long sequencing reads. PLoS Comput Biol. 2017 Jun 8;13(6):e1005595. doi: 10.1371/journal.pcbi.1005595. PMID: 28594827; PMCID: PMC5481147.

11. Lin Y, Yuan J, Kolmogorov M, Shen MW, Chaisson M, Pevzner PA. Assembly of long error-prone reads using de Bruijn graphs. Proc Natl Acad Sci U S A. 2016 Dec 27;113(52):E8396-E8405. doi: 10.1073/pnas.1604560113. Epub 2016 Dec 12. PMID: 27956617; PMCID: PMC5206522.

12. Vaser R, Sović I, Nagarajan N, Šikić M. Fast and accurate de novo genome assembly from long uncorrected reads. Genome Res. 2017 May;27(5):737-746. doi: 10.1101/gr.214270.116.Epub 2017 Jan 18. PMID: 28100585; PMCID: PMC5411768.

13. Lee JY, Kong M, Oh J, Lim J, Chung SH, Kim JM, Kim JS, Kim KH, Yoo JC, Kwak W. Comparative evaluation of Nanopore polishing tools for microbial genome assembly and polishing strategies for downstream analysis. Sci Rep. 2021 Oct 20;11(1):20740. doi: 10.1038/s41598-021-00178-w. PMID: 34671046; PMCID: PMC8528807

14. Walker BJ, Abeel T, Shea T, Priest M, Abouelliel A, Sakthikumar S, Cuomo CA, Zeng Q,Wortman J, Young SK, Earl AM. Pilon: an integrated tool for comprehensive microbial variant detection and genome assembly improvement. PLoS One. 2014 Nov19;9(11):e112963. doi: 10.1371/journal.pone.0112963. PMID: 25409509; PMCID: PMC4237348.

15. Liu CM, Stegger M, Aziz M, Johnson TJ, Waits K, Nordstrom L, Gauld L, Weaver B, Rolland D, Statham S, Horwinski J, Sariya S, Davis GS, Sokurenko E, Keim P, Johnson JR,Price LB. Escherichia coli ST131-H22 as a Foodborne Uropathogen. mBio. 2018 Aug. doi: 10.1128/mBio.00470-18. 28;9(4):e00470-18.

16. Seemann T. Prokka: rapid prokaryotic genome annotation. Bioinformatics. 2014 Jul 15;30(14):2068-9. doi: 10.1093/bioinformatics/btu153. Epub 2014 Mar 18. PMID: 24642063.

17. Page AJ, Cummins CA, Hunt M, Wong VK, Reuter S, Holden MT, Fookes M, Falush D,Keane JA, Parkhill J. Roary: rapid large-scale prokaryote pan genome analysis. Bioinformatics. 2015 Nov 15;31(22):3691-3. doi: 10.1093/bioinformatics/btv421. Epub 2015 Jul 20. PMID: 26198102; PMCID: PMC4817141.

18. Stamatakis A. RAxML version 8: a tool for phylogenetic analysis and post-analysis of large phylogenies. Bioinformatics. 2014 May 1;30(9):1312-3. doi: 10.1093/bioinformatics/btu033.Epub 2014 Jan 21. PMID: 24451623; PMCID: PMC3998144.

19. Letunic I, Bork P. Interactive Tree Of Life (iTOL) v4: recent updates and new developments.Nucleic Acids Res. 2019 Jul 2;47(W1):W256-W259. doi: 10.1093/nar/gkz239. PMID: 30931475; PMCID: PMC6602468.

20. Zelendova M, Papagiannitsis C, Sismova P, Medvecky M, Pomorska K, Palkovicova J,Jakubu V, Jamborova I, Zemlickova H, Dolejska M, Working Group for Monitoring ofAntibiotic Resistance. Plasmid-mediated colistin resistance among human clinical Enterobacterales isolates: National surveillance in the Czech Republic.BIORXIV/2023/527831.

21. Zelendova M, Papagiannitsis CC, Valcek A, Medvecky M, Bitar I, Hrabak J, Gelbicova T, Barakova A, Kutilova I, Karpiskova R, Dolejska M. Characterization of the Complete Nucleotide Sequences of *mcr-1*-Encoding Plasmids From Enterobacterales Isolates in Retailed Raw Meat Products From the Czech Republic. Front Microbiol. 2021 Jan 15;11:604067. doi: 10.3389/fmicb.2020.604067. PMID: 33519748; PMCID: PMC7843963.

22. Tarabai H, Valcek A, Jamborova I, Vazhov SV, Karyakin IV, Raab R, Literak I, Dolejska M. Plasmid-Mediated *mcr-1* Colistin Resistance in Escherichia coli from a Black Kite in Russia. Antimicrob Agents Chemother. 2019 Aug 23;63(9):e01266-19. doi: 10.1128/AAC.01266-19. PMID: 31307988; PMCID: PMC6709458.

23. Alba P, Leekitcharoenphon P, Franco A, Feltrin F, Ianzano A, Caprioli A, Stravino F, Hendriksen RS, Bortolaia V, Battisti A. Molecular Epidemiology of *mcr*-Encoded Colistin Resistance in *Enterobacteriaceae* From Food-Producing Animals in Italy Revealed Throughthe EU Harmonized Antimicrobial Resistance Monitoring. Front Microbiol. 2018 Jun 12;9:1217. doi: 10.3389/fmicb.2018.01217. PMID: 29951045; PMCID: PMC6008537.

24. Liu YY, Wang Y, Walsh TR, Yi LX, Zhang R, Spencer J, Doi Y, Tian G, Dong B, Huang X, Yu LF, Gu D, Ren H, Chen X, Lv L, He D, Zhou H, Liang Z, Liu JH, Shen J. Emergence of plasmid-mediated colistin resistance mechanism MCR-1 in animals and human beings in China: a microbiological and molecular biological study. Lancet Infect Dis. 2016 Feb;16(2):161-8. doi: 10.1016/S1473-3099(15)00424-7. Epub 2015 Nov 19. PMID: 26603172.

25. Xavier BB, Lammens C, Ruhal R, Kumar-Singh S, Butaye P, Goossens H, Malhotra-Kumar S.Identification of a novel plasmid-mediated colistin-resistance gene, mcr-2, in Escherichia coli,Belgium, June 2016. Euro S urveill. 2016 Jul 7;21(27). doi: 10.2807/1560- 7917.ES.2016.21.27.30280. Erratum in: Euro Surveill. 2016 Jul 14;21(28): PMID: 27416987.

26. Yin W, Li H, Shen Y, Liu Z, Wang S, Shen Z, Zhang R, Walsh TR, Shen J, Wang Y. NovelPlasmid-Mediated Colistin Resistance Gene mcr-3 in Escherichia coli. mBio. 2017 Jun 27;8(3):e00543-17. doi: 10.1128/mBio.00543-17. Erratum in: MBio. 2017 Aug 15;8(4): PMID: 28655818; PMCID: PMC5487729.

27. Carattoli A, Villa L, Feudi C, Curcio L, Orsini S, Luppi A, Pezzotti G, Magistrali CF. Novelplasmid-mediated colistin resistance mcr-4 gene in Salmonella and Escherichia coli, Italy 2013, Spain and Belgium, 2015 to 2016. Euro Surveill. 2017 Aug 3;22(31):30589. doi: 10.2807/1560-7917.ES.2017.22.31.30589. PMID: 28797329; PMCID: PMC5553062.

28. Rebelo, AR, Bortolaia, V, Kjeldgaard, JS, Pedersen, SK, Leekitcharoenphon, P, Hansen, IM,Guerra, B, Malorny, B, Borowiak, M, Hammerl, JA, Battisti, A, Franco, A, Alba, P, Perrin- Guyomard, A, Granier, SA, De Frutos Escobar, C, Malhotra-Kumar, S, Villa, L, Carattoli, A,Hendriksen, RS. Multiplex PCR for detection of plasmid-mediated mcr-4 and mcr-5 for surveillance purposes. Euro surveill. 2018, 23, pii=17-00672. doi:/10.2807/1560- 7917.ES.2018.23.6.17-00672

29. AbuOun M, Stubberfield EJ, Duggett NA, Kirchner M, Dormer L, Nunez-Garcia J, Randall LP, Lemma FL, Crook DW, Teale C, Smith RP, Anjum MF. mcr-1 and mcr-2 (mcr-6.1) variantgenes identified in Moraxella species isolated from pigs in Great Britain from 2014 to 2015, Journal of Antimicrobial Chemotherapy, Volume 72, Issue 10, October 2017, Pages 2745–2749, https://doi.org/10.1093/jac/dkx286

30. Wang X, Wang Y, Zhou Y, Li J, Yin W, Wang S, Zhang S, Shen J, Shen Z, Wang Y. Emergence of a novel mobile colistin resistance gene, mcr-8, in NDM-producing Klebsiella
pneumoniae. Emerg Microbes Infect. 2018 Jul 4;7(1):122. doi: 10.1038/s41426-018-0124-z.PMID: 29970891; PMCID: PMC6030107.

31. Kieffer N, Royer G, Decousser JW, Bourrel AS, Palmieri M, Ortiz De La Rosa JM, Jacquier H, Denamur E, Nordmann P, Poirel L. mcr-9, an Inducible Gene Encoding an Acquired Phosphoethanolamine Transferase in Escherichia coli, and Its Origin. Antimicrob Agents Chemother. 2019 Aug 23;63(9):e00965-19. doi: 10.1128/AAC.00965-19. Erratum in: Antimicrob Agents Chemother. 2019 Oct 22;63(11): PMID: 31209009; PMCID: PMC6709461.

32. Wang C, Feng Y, Liu L, Wei L, Kang M, Zong Z. Identification of novel mobile colistinresistance gene mcr-10. Emerg Microbes Infect. 2020 Mar 2;9(1):508-516. doi: 10.1080/22221751.2020.1732231. PMID: 32116151; PMCID: PMC7067168.
